# Application of Nanocomposites in Covalent Organic Framework-Based Electrocatalysts

**DOI:** 10.3390/nano14231907

**Published:** 2024-11-27

**Authors:** Haiping Zhou, Kechang Li, Qingqing Pan, Zhongmin Su, Rui Wang

**Affiliations:** 1 State Key Laboratory of Inorganic Synthesis and Preparative Chemistry, The institute of Theoretical Chemistry, College of Chemistry, Jilin University, Changchun 130012, Chinazmsu@nenu.edu.cn (Z.S.); 2 School of Chemistry and Environmental Engineering, Jilin Provincial Science and Technology Innovation Center of Optical Materials and Chemistry, Changchun University of Science and Technology, Changchun 130012, China

**Keywords:** covalent organic framework, electrocatalysis, nanocomposites

## Abstract

In recent years, the development of high-performance electrocatalysts for energy conversion and environmental remediation has become a topic of great interest. Covalent organic frameworks (COFs), linked by covalent bonds, have emerged as promising materials in the field of electrocatalysis due to their well-defined structures, high specific surface areas, tunable pore structures, and excellent acid–base stability. However, the low conductivity of COF materials often limits their intrinsic electrocatalytic activity. To enhance the catalytic performance of COF-based catalysts, various nanomaterials are integrated into COFs to form composite catalysts. The stable and tunable porous structure of COFs provides an ideal platform for these nanomaterials, leading to improved electrocatalytic activity. Through rational design, COF-based composite electrocatalysts can achieve synergistic effects between nanomaterials and the COF carrier, enabling efficient targeted electrocatalysis. This review summarizes the applications of nanomaterial-incorporated COF-based catalysts in hydrogen evolution, oxygen evolution, oxygen reduction, carbon dioxide reduction, and nitrogen reduction. Additionally, it outlines design principles for COF-based composite electrocatalysis, focusing on structure–activity relationships and synergistic effects in COF composite nanomaterial electrocatalysts, as well as challenges and future perspectives for next-generation composite electrocatalysts.

## 1. Introduction

In the past decade, the widespread use of fossil fuels has posed a huge challenge to the sustainable use of the environment and energy that we depend on for our livelihood [1]. Electrocatalysis is an indispensable means to produce clean energy (such as hydrogen) and convert atmospheric water, carbon dioxide, oxygen, etc., into high value-added products, and even energy storage devices such as fuel cells and metal–air batteries [2]. However, it is difficult to effectively determine the structure of many electrocatalysts so far and then explore their potential structure–activity relationships [3]. The existing catalysts are not only constrained by slow reaction kinetics, but also not conducive to exploring the potential mechanism of catalysts with clear structure–activity relationships [4]. Therefore, in order to overcome this problem, there is an urgent need to develop advanced catalysts with well-defined structures, high catalytic activity, and selectivity [4].

The covalent organic framework (COFs) has attracted extensive attention from researchers in the field of electrocatalysis due to its highly ordered and well-defined crystal structure, tunable abundant pore structure, and high specific surface area [5]. The crystalline polymers composed of light elements connected by covalent bonds can be pre-designed to provide a large number of periodic active sites for the electrocatalytic process and promote the reaction energy barrier and reaction in the electrocatalytic process [6], although other porous materials such as metal–organic frameworks (MOFs) and covalent–organic polymers (COPs) can be designed to achieve similar effects [7]. However, the intrinsic structural instability of MOF materials limits the development of their long-term durability as electrocatalysts. Moreover, COPs lack a periodic ordered crystal structure, which cannot clearly explain the structure–activity relationship between the structure and performance of electrocatalysts, and is not conducive to the leakage of sufficient active sites [8]. Therefore, the development of COF-based electrocatalysts with high catalytic activity has become an urgent problem to be solved [8].

Although some fruitful progress has been made in COF-based electrocatalysts, it is difficult for COF-based catalysts to be used as electrocatalysts alone without adding other additives due to their inherent low electronic conductivity, difficult to have a catalytic performance comparable to that of metal-based catalysts, and they have poor long-term durability. Until now, nanomaterials have been added to a wide variety of electrocatalysts as effective additives [9,10]. Various metal nanomaterials (metal ions, metal nanoparticles, metal compounds, etc.) and carbon nanomaterials (including graphene and carbon nanotubes) have been explored as effective additives that can greatly improve the performance of catalysts [11]. It is worth noting that these nano-additives have also been successfully demonstrated to be an effective means to improve the catalytic performance of COFs-based materials in COFs [12]. The COF-based catalysts of this composite nanomaterial are emerging in the fields of hydrogen evolution reactions (HERs), oxygen evolution reactions (OERs), oxygen reduction reactions (ORRs), hydrogen oxidation reactions (HORs), nitrogen reduction reactions (NRRs), and carbon dioxide reduction reactions (CO_2_RRs) [13]. However, there is no relevant review on the development of catalysts for COF-based nanomaterial composites. In this review, we introduce the design principles of COF-based nano-catalysts and the structural properties and synergistic effects of the catalysts. It also reviewed the current state of development in this field. Through the recent experimental research work, the current progress of these nanocomposite COF-based catalysts is highlighted, and some unsolved difficulties with COF-based electrocatalysts are proposed, as well as the challenges and research goals faced. We believe that this study will provide some enlightenment for the design of COFs-based electrocatalysts with well-defined structures and the study of structure–activity relationships.

## 2. Application Prospects of COF-Based Nanocomposite Catalysts

### 2.1. Structure and Characteristics of COF

**Figure 1:**
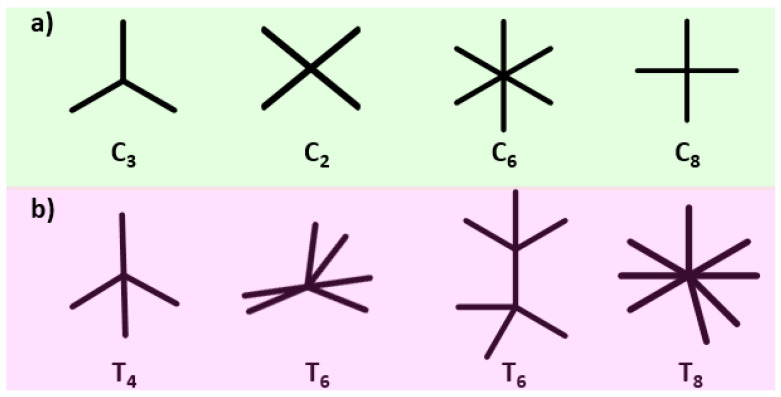
Universal linker for (**a**) 2D COFs and (**b**) 3D COFs.

COFs are a type of crystalline organic polymer characterized by a regular pore structure, interconnected by covalent bonds, and composed of light elements. Since the Yaghi group first reported 2D COFs—COF-1 and COF-5—connected via boron ester linkages in 2005, significant advancements have been made in this field [14]. Currently, 2D COFs have emerged as the mainstream in the field of COFs due to their more extensive variety of organic building units and simpler synthesis compared to 3D COFs as shown in Figure 1. [15]. In 2D COFs, covalent bonds are confined to the conjugated two-dimensional layered structures, while weak interactions, such as π–π stacking, hydrogen bonding, and van der Waals forces, primarily occur between the layers [16]. In contrast, the entire framework of 3D COFs is interconnected by covalent bonds [17]. Typically, 2D COFs feature uniform one-dimensional channels, whereas 3D COFs exhibit more complex interpenetrating channels that enhance their suitability for applications such as separation, catalysis, and guest binding [18]. Moreover, when compared to 2D structures, 3D COFs generally possess higher surface areas, lower densities, and a greater abundance of accessible active sites, making them particularly advantageous for practical catalysis and gas adsorption [19]. On the other hand, the stability of 2D COFs is often superior to that of 3D COFs due to a reduced number of empty frameworks and the absence of π–π stacking interactions [20]. Additionally, interpenetration is commonly observed in 3D networks, especially in dia or pts topologies, leading to highly constricted channels. This characteristic often complicates the loading of nanomaterials into the pores of 3D COFs. To date, various types of covalent bonds have been successfully employed in COFs, including nitrogen-containing linkers (such as imides, hydrazides, triazines, ureas, benzenoid imines, amides, imino amides, diazine dioxide, etc.), boron-containing linkers (boroxines, boronic esters, spiroborate salts, and borosilicate salts), heterocyclic linkers (imidazoles, oxazoles, thiazoles, etc.), and alkyl linkers (ethylene, ester linkages, polyphenylene ethers, etc.) [21].

**Figure 2:**
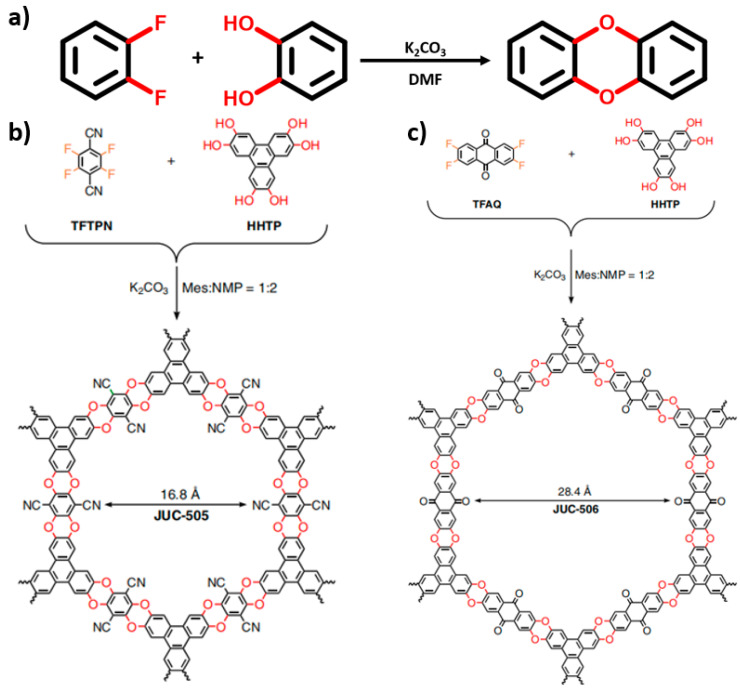
(**a**) Schematic diagram of the formation of new linkers.Design and structure of JUC-505 (**b**) and JUC-506 (**c**) COF based on polarly ether linkage. Reproduced with permission from ref. [16], Copyright 2019, from Springer Nature.

Notably, as shown in Figure 2, our group reported a multi-phenyl ether-linked COF in 2019 [22]. These COFs demonstrated exceptional durability, surpassing all previously known varieties of COFs. Currently, the stability of these covalent bonds as linkers is stronger than the coordination bonds present in metal–organic frameworks (MOFs) and the hydrogen bonds found in organic frameworks (HOFs), particularly in terms of long-term durability in acidic or alkaline aqueous solutions. This robustness lays a solid foundation for the extensive application of COF-based materials in electrocatalysis.

**Figure 3:**
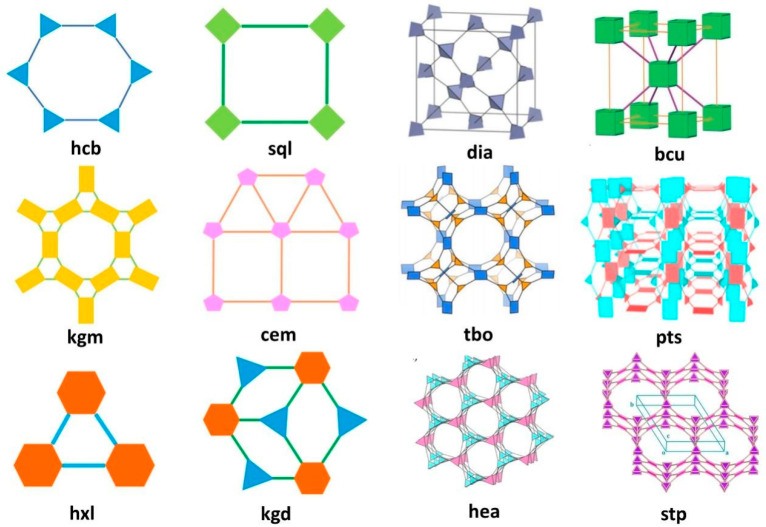
Common 2D and 3D COFs topologies reported in the literature so far.

Topology is another crucial characteristic of COF materials. As COFs are constructed from small organic molecules of varying geometric shapes interconnected by covalent bonds, they can yield numerous topologies based on the distinct geometric configure rations and spatial arrangements of their building blocks, as well as their various connection modes [23]. Figure 3 illustrates some common topologies found in COFs. For two-dimensional COFs, prevalent topologies include hcb, sql, hxl, kgd, kgm, and tth. In contrast, three-dimensional COFs typically exhibit more complex topologies. To date, over 30 different topologies of three-dimensional COFs have been documented [24]. The diversity in topology significantly impacts their pore structures. On one hand, three-dimensional COFs may possess interpenetrating structures, leading to generally smaller pore sizes compared to those of two-dimensional COFs. On the other hand, their complex spatial arrangements result in a higher specific surface area relative to two-dimensional counterparts. Rigid and uniform pores can effectively support nanomaterials in COF-based electrocatalysts. Although COFs demonstrate greater stability than zeolites, their pore size can limit their effectiveness as supports for nanomaterials in electrocatalytic applications. By fine-tuning the size and shape of the building units, it is possible to design COF pores in a targeted manner, enabling the accommodation of various nanomaterials and facilitating the formation of homogeneous materials. COFs typically exhibit microporous or mesoporous characteristics, thereby promoting the diffusion of electrolytes in electrocatalytic reactions and enhancing mass transfer processes [25].

### 2.2. COF-Based Electrocatalysts for Nanomaterial Composites

**Figure 4:**
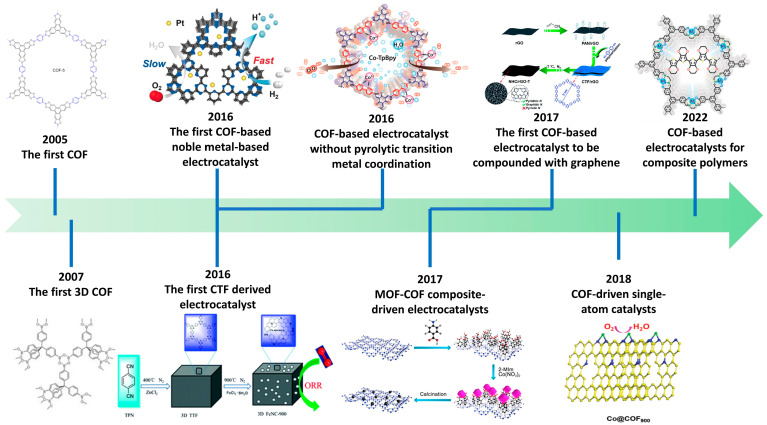
Development of COFs-based electrocatalysts for nanomaterial composites.

Since 2005, when Yaghi’s group developed COFs, different types of COF-based electrocatalysts have been developed in view of the rapid development of the field of electrocatalysis for MOFs. At present, the strategies for constructing electrocatalysts using similar materials can be divided into the pyrolysis method, defect engineering method, heterojunction engineering method, interfacial programming method, and nanomaterial filling method as shown in Figure 4 [26]. COF-filled nanocomposites differ significantly from MOF-derived or COF-derived materials and electrocatalysts developed through defect engineering, heterojunction engineering, and interface engineering strategies [27]. These nanocomposites are built on highly ordered covalent crystal structures, providing exceptional chemical stability and design versatility. The integration of nanomaterials enhances the catalytic performance through synergistic effects, while their structure and properties can be easily tailored during the filling process. Moreover, their preparation is energy-efficient, environmentally friendly, and preserves the material’s ordered framework. In contrast, MOF-derived or COF-derived materials rely on high-temperature pyrolysis to produce multifunctional active sites, such as metal oxides, phosphides, or carbides [28]. While these materials typically offer high surface areas, the pyrolysis process can lead to structural degradation, reducing stability. Defect engineering optimizes electronic structures by introducing lattice defects, but may compromise material stability. Heterojunction and interface engineering improve catalytic efficiency by enhancing interface reactions and the charge transfer, yet these approaches often involve complex synthesis and precise control [29]. Overall, COF-filled nanocomposites excel in terms of uniform active site distribution, efficient charge transport, environmental sustainability, and structural stability, making them a promising and sustainable solution for advanced electrocatalyst design.

For COF-based electrocatalysts combined with nanomaterials, the catalytic efficiency of COF-based catalysts at the molecular level is strongly influenced by charge transfer processes, interactions with nanomaterials, and their collective impact on reaction dynamics. The ordered porous structure of COFs provides an ideal platform for an efficient charge transfer, enabling the adsorption, activation, and desorption of reactants and products. Modifying the COF framework, such as incorporating metal centers or functional groups, enhances the charge mobility and reduces reaction barriers [30]. Furthermore, the synergy between COFs and nanomaterials—such as metal nanoparticles or carbon-based materials—introduces additional active sites, optimizes electron transfer pathways, and significantly improves the conductivity and catalytic performance [31]. These interactions not only accelerate catalytic kinetics by lowering the activation energy, but also enhance the stability under harsh conditions and enable the precise tuning of reaction pathways for improved selectivity. Together, these attributes make COF-based catalysts highly effective for energy conversion and other catalytic applications.

COF modification effectively reduces the energy barriers in various electrochemical reactions by optimizing the structure and active site distribution. Its highly ordered crystal structure and porous nature accelerate reactant transport, while the introduction of functional groups or filling materials precisely tunes the electronic structure of the active sites, further enhancing the catalytic performance through synergistic effects. Additionally, the optimization of surface functional groups and interface structures improves the adsorption and desorption efficiency of intermediates, while conductivity enhancements facilitate the charge transfer [32]. Through these mechanisms, COF modification provides lower energy barriers and more efficient reaction pathways for electrochemical reactions such as ORR, OER, and HER.

COF-based electrocatalysts reduce energy barriers in various electrochemical reactions, including ORR, OER, HER, HOR, NRR, and CO_2_RR, through a combination of advanced design strategies. Their ordered porous structure promotes efficient reactant adsorption, diffusion, and activation, while the incorporation of transition metals (e.g., Fe, Co, and Ni) creates highly active metal-N coordination sites that significantly lower the activation energy [33]. An enhanced charge transfer, supported by π–π conjugation and functionalized COF frameworks, improves the electron mobility and reaction kinetics. Synergistic integration with nanomaterials boosts conductivity and introduces additional active sites, while defect engineering generates unsaturated sites to strengthen intermediate adsorption. Functional modifications further optimize reactant interactions, stabilize intermediates, and refine reaction pathways [34]. Together, these features enable COF-based catalysts to achieve lower overpotentials, improved selectivity, and high catalytic efficiency across a wide range of reactions.

### 2.3. Types of Nanocomposites Used in COF-Based Electrocatalysts

Due to the rich and controllable pore structure of COFs, various types and sizes of nanomaterials can be incorporated into their pores [35]. This loading process not only involves physical mixing, but also indicates that the two components form an inseparable composite system. To date, the incorporation of nanomaterials into COFs has been successfully applied in diverse fields such as gas separation, photocatalysis, electrocatalysis, energy storage, and sensing. Among the various nanomaterials, metal nanomaterials are the most widely utilized [36]. Currently, metal nanomaterials, including metal ions, metal nanoparticles, and metal oxides or sulfides, have been supported in COFs through different methods [37].

The loading process can be categorized into the pre-loading and post-synthesis modification of COFs. Pre-loading involves synthesizing small organic molecules containing metals, which are then employed as building blocks in the synthesis of COF materials. Typical metal-containing small organic molecules that can be pre-loaded include porphyrins and phthalocyanines, which are extensively used in constructing COF catalysts for electrocatalysis. Multiple nitrogen-rich COFs have been developed using components or linkers incorporating nitrogen sources such as imines and triazines to provide coordination sites for metals [38]. Following synthesis, these COFs are often coordinated with metal salts, which are subsequently reduced to form metal nanoparticles or other metal nanomaterials, using reducing agents or thermal decomposition as needed.

In addition to metal nanomaterials, carbon nanomaterials are also widely employed in COF-based catalysts to create composite nanomaterials [39]. Carbon nanomaterials—including 0D carbon quantum dots, 1D carbon nanotubes, and 2D graphene—have been shown to significantly enhance the catalytic performance of electrocatalysts. These carbon nanomaterials exhibit excellent electronic conductivity, making them ideal complements to COFs (which act as semiconductor materials). Typically, these carbon nanomaterials are combined with COFs through physical mixing and primarily serve as conductive agents. Recent studies have reported the blending of these two types of materials into a homogeneous entity through physical mixing followed by pyrolysis, thereby fully utilizing their respective advantages [40]. Compared to heterogeneous materials, this approach helps to mitigate catalyst aggregation, thereby enhancing the stability of electrocatalysts [41].

Another significant category of nanofillers includes other porous materials. Reports have indicated that MXene and MOFs (such as ZIF) can be used directly or indirectly as COF-based electrocatalysts. Modifying organic functional groups on ligands allows for the in situ growth of MOFs [42]. MXene itself can act as an effective electrocatalyst, and when integrated into COFs, the confinement effect of COFs can significantly enhance its catalytic performance [43]. Additionally, polyoxometalates (POMs) are nano-scale metal–oxygen cluster compounds composed of high oxidation state transition metal ions and oxygen. In COF-based catalysts, COFs can serve as guest fillers [42].

Recently, organic molecules, including organic polymers, have been utilized as carriers in COF-based composite materials. Conductive polymers such as polypyrrole and poly(3,4-ethylenedioxythiophene) (PEDOT) possess excellent intrinsic electronic conductivity, significantly improving the conductivity of composite catalysts [44]. Moreover, pure organic structures can establish supramolecular interactions with host COFs, thereby inhibiting aggregation phenomena and facilitating further doping. By co-pyrolyzing organic molecules rich in elements like nitrogen, sulfur, and phosphorus with COFs, non-metallic catalysts doped with multiple heteroatoms can be synthesized [45]. The investigation of nanomaterial reorganization in COF-based catalysts based on these strategies will be discussed in detail in the next section.

## 3. Recent Advances in Hybrid Nanomaterials and Nanocomposites in Covalent Organic Framework-Based Electrocatalysts

### 3.1. Covalent Organic Framework-Based Electrocatalysts for the Recombination of Nano Precious Metallic Materials

Precious metals, including platinum, palladium, gold, ruthenium, and iridium, have been shown to play a significant role in various electrocatalytic processes, such as hydrogen conversion, carbon dioxide reduction, and nitrogen reduction [46]. These metals have been successfully integrated into COFs, facilitating the construction of effective catalysts for electrocatalytic water splitting, ORR, and HOR [47].

**Figure 5:**
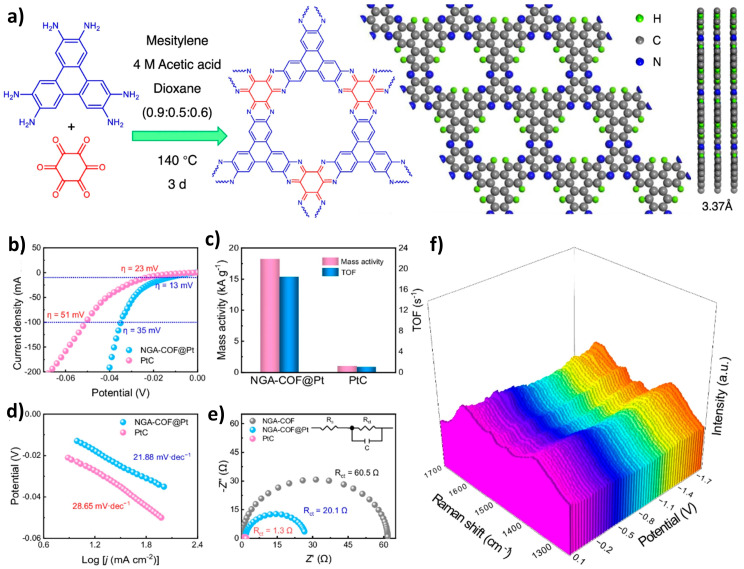
(**a**) Schematic diagram of NGA-COF synthesis. (**b**) Polarization curves for different samples, (**c**) mass activity and TOF, (**d**) corresponding Tafel plots, and (**e**) EIS (inset shows equivalent circuit diagrams). (**f**) In situ Raman spectra of NGA at various potentials COF@Pt. Reproduced with permission from ref. [51], Copyright 2024, from Nature Springer.

Platinum, recognized for its efficiency in both the HER and ORR, has been effectively introduced as a nanofiller in COFs-based electrocatalysts. In 2016, Kamai first utilized nitrogen-coordinated platinum ions within covalent triazine frameworks (CTFs), known as Pt-CTF, for HOR reactions [48]. Notably, even with a platinum support content of only 3 wt%, the Pt-CTF catalyst exhibited HOR activity comparable to that of a Pt/C catalyst containing 20 wt% platinum. COFs loaded with platinum have demonstrated effectiveness for both HER and HOR applications. For instance, in 2020, Park et al. designed and synthesized COF-bpyTPP, derived from 4-aminophenyl porphyrin and 4,4-dialdehyde-based pyridine building blocks, showcasing a significantly lower starting potential of 0.13 V compared to a commercial Pt/C catalyst (0.65 V) [49]. Additionally, Wang et al. reported a COF that simultaneously incorporated Co_3_O_4_ and platinum, achieving a low overpotential of 23 mV at a current density of 10 mA cm^−2^ for HER [50]. In the same year, our research group developed a nitrogen-rich graphene analogue COF (NGA-COF) that embedded platinum single atoms through electrochemical modification, serving as a HER catalyst without the need for additional conductive agents or pyrolysis treatment as shown in Figure 5 [51]. This platinum single-atom catalyst, characterized by its unique Pt-N_2_ coordination environment, required only 13 mV of overpotential at a current density of 10 mA cm^−2^, positioning it as one of the most efficient HER catalysts reported to date. Further noteworthy advancements include the design and synthesis of a Pt-supported COF (Pt@TTB-COF) by Fan et al. in 2024. This COF, built on a triazine framework, demonstrated an exceptional HER performance with supported platinum single atoms, achieving an impressive overpotential of just 5 mV at a current density of 10 mA cm^−2^ [52]. Yang’s group also contributed significantly by developing an amino-functionalized COF in 2024, which served as a high-efficiency HER catalyst post-platinum coordination, showing an overpotential of 19 mV at a current density of 10 mA cm^−2^ [53].

In the realm of ORR, Zhai et al. achieved efficient catalysis by uniformly loading platinum nanoparticles into a COF with triazine structural units in 2020 [54]. This COF featured multiple pyridine nitrogen sites that acted as nucleation points for platinum nanoparticles, allowing for controlled and uniform growth on both the COF surface and its pores, resulting in a half-wave potential of up to 0.89 V as an ORR electrocatalyst. Building on this momentum, subsequent studies in 2022 and beyond have further explored the potential of COF-based electrocatalysts loaded with platinum in ORR applications. Research by Zhai et al. utilized COF-300 with platinum, derived from pyrolysis using porous nitrogen-doped carbon from POFs, which resulted in an ORR electrocatalyst exhibiting a half-wave potential of up to 0.85 V [55]. Following this, efforts included the pyrolysis of TP–BPY–COF with platinum loading at 800 °C, yielding an ORR electrocatalyst with a half-wave potential of up to 0.88 V [56]. Additionally, Wang’s group employed the SiO_2_ template method to pyrolyze 2D TpPa-COF loaded with platinum, producing nitrogen-doped porous carbon containing homogeneous ultrafine platinum nanoparticles, achieving an impressive overpotential as high as 0.93 V when employed as an ORR electrocatalyst [57].

Ruthenium dioxide is a widely recognized and commercially available electrocatalyst for OER, renowned for its high efficiency. Recently, ruthenium-loaded COFs have also demonstrated remarkable performance in OER applications. In 2019, the Vaidhyanathan team developed two types of ruthenium-containing COFs that were directly used as OER electrocatalysts after undergoing pyrolysis, achieving overpotentials below 220 mV at a current density of 10 mA cm^−2^ [58]. In 2022, Zhao et al. reported a copper and ruthenium dual-metal-supported COF derivative, which served as a bifunctional electrocatalyst for HER and OER through a combination of ion exchange and the PS template-assisted pyrolysis technique [59].

In 2023, another research team incorporated ruthenium ions into a melamine-based COF, which was then converted into porous carbon through pyrolysis and utilized as a catalyst for overall water splitting. This catalyst exhibited an excellent performance with an overpotential of 9 mV for HER and 280 mV for OER, at a current density of 10 mA cm^−2^ [60]. Ruthenium also showed unique advantages as a HER catalyst. In 2019, Sun et al. pyrolyzed a nitrogen-rich iron-coordinated COF, using it directly as a HER catalyst in acidic and alkaline electrolytes, achieving overpotentials of only 7 mV (0.5 M H_2_SO_4_) and 7 mV (1.0 M KOH) at a current density of 10 mA cm^−2^ [61]. Similarly, the Das team reported a two-dimensional COF containing ruthenium ions that could be directly employed as a HER catalyst in acidic electrolytes without the need for pyrolysis treatment, with an initial potential as low as 159 mV [62].

In 2021, Pan et al. uniformly loaded ruthenium nanoclusters into a COF constructed from melamine units. The resulting catalyst PMDA/MA-Ru-180-800 achieved an impressive overpotential of only 35.1 mV at a current density of 10 mA cm^−2^ after pyrolysis [63]. The following year, Chen et al. designed and synthesized a pyridyl-rich conjugated COF, constructing a ruthenium-supported HER electrocatalyst (Ru@COF-1) using a unique coordination method (Ru-N_2_Cl_2_), which achieved an overpotential of 200 mV at a current density of 10 mA cm^−2^ [64]. Additionally, ruthenium-loaded COFs showed potential in electrocatalytic NRR. Recently, Shetty’s team reported a pyridine-rich ruthenium-loaded COF as an NRR electrocatalyst, maintaining a Faradaic efficiency (FE) exceeding 50% and a productivity of over 2 mg h^−1^ mg_cat_^−1^ [65]. They also applied this catalyst to electrocatalytic nitrate reduction reactions (NO_3_RR) for ammonia production, maintaining an excellent FE of over 90% and a productivity of 1.16 mg h^−1^ cm^−2^ [66].

Other noble metals, including gold, silver, palladium, and rhodium, have also been incorporated into COFs as electrocatalysts. Guo et al. recently synthesized a highly conjugated COF containing a heptazine building block, where the abundance of pyridyl nitrogen provided coordination sites for gold. The resulting M−HCO−CTF@AuNPs served as an NRR electrocatalyst, achieving an ammonia yield of 66.3 μg h^−1^ mg^−1^ [67]. In 2019, the Janiak team utilized rhodium nanoparticles on CTF-1 as an efficient HER electrocatalyst, requiring only an overpotential of 58 mV to achieve a current density of 10 mA cm^−2^ [68]. In 2022, Yue et al. reported the first palladium-supported HER electrocatalyst based on COFs (PY-SE–COF–Pd), which achieved an overpotential of 128 mV at a current density of 10 mA cm^−2^ [69].

In 2023, Feng et al. pre-loaded palladium nanoparticles into the pores of a three-dimensional COF (TB-COF) and obtained an ORR electrocatalyst through pyrolysis, attaining a half-wave potential of up to 0.906 V [70]. In the same year, Liu’s team grew palladium nanoparticles in situ within a pre-synthesized WTA-COF, which was constructed from triphenylamine units, serving as an ORR catalyst with a half-wave potential reaching 0.865 V [71]. Additionally, in 2021, Wang et al. designed and synthesized a metal covalent organic framework (MCOF) containing cobalt coordinated with bipyridyl functional groups, incorporating Ag/AgO into the MCOF to directly serve as an ORR electrocatalyst, achieving a half-wave potential of up to 0.76 V in alkaline electrolytes [72].

COF-based noble metal electrocatalysts offer significant advantages, including highly ordered structures and large surface areas, which help evenly distribute noble metal active sites and enhance the catalytic efficiency. Their good designability allows for the flexible tuning of the structure, pore size, and functional groups to further optimize the performance. The synergistic effect with noble metals can reduce the amount of noble metal used, improving both economic feasibility and sustainability. Additionally, COFs possess high chemical stability and low energy consumption, making them environmentally friendly. However, there are also some drawbacks, such as the still relatively high usage of noble metals, which increases the cost, especially for large-scale applications. The limitations of the COF pore structure may hinder the transport of reactants, thus affecting the catalytic efficiency. Furthermore, the synthesis process of COFs is complex, raising preparation costs and technical requirements, and the noble metal component may detach or aggregate during use, leading to catalyst deactivation.

### 3.2. Covalent Organic Framework-Based Electrocatalysts for the Recombination of Transition Metallic Materials

Compared to precious metals, transition metal catalysts offer abundant reserves and cost advantages, along with robust redox properties within the d-electron layer, making them susceptible to electron loss or capture [73]. Consequently, they are considered the most promising type of catalyst in current research.

Jin et al. designed and synthesized an NRR catalyst featuring a titanium–porphyrin structure that effectively suppresses HER while enhancing the activity of nitrogen molecules, achieving over 25 μg h^−1^ mg_cat_^−1^ and an FE exceeding 34% [74]. Similarly, in 2024, Mishra et al. developed an anthraquinone-based NRR electrocatalyst loaded with TiO_2_, demonstrating a high ammonia production rate of 30 μg mg^−1^ h^−1^ while maintaining an FE of about 16% [75].

In 2021, Filo’s group introduced a COF featuring manganese single atoms supported by bipyridine as an electrocatalyst for carbon dioxide reduction reactions (CO_2_RR) [76]. This catalyst exhibited a starting potential of around 190 mV and a current density of up to 12 mA cm^−2^ at an overpotential of 550 mV. Yue et al. synthesized a series of metalloporphyrin-based COFs as ORR electrocatalysts in 2021, with the manganese-loaded COF (M-TP-COFs) achieving a high half-wave potential of 0.75 V, significantly higher than the unloaded metal-loaded porphyrin-based COFs, which had a half-wave potential of approximately 0.71 V [77].

In 2020, Luo’s group developed a cobalt and vanadium bimetallic OER electrocatalyst supported by sulfonic acid. By adjusting the ratios of cobalt and vanadium, they produced Co_0.5_V_0.5_@COF-SO_3_, a catalyst demonstrating catalytic activity comparable to commercial IrO_2_ catalysts [78]. Zhang et al. (2019) created a new covalent porphyrin framework (CPFs) supported by FeSn_2_@FeSnO_x_ as an ORR electrocatalyst through pyrolysis, showing an excellent catalytic performance under both acidic and alkaline conditions—especially in alkaline media, with a half-wave potential of up to 0.88 V, outperforming commercial Pt/C catalysts [79]. Qiao’s team prepared ORR electrocatalysts from copper–tin ultrafine nanoalloys derived from COFs, achieving a half-wave potential of 0.86 V in alkaline electrolytes [80].

Molybdenum-based metal compounds have also gained attention as effective nanocomposites for electrocatalysis. In 2019, Hu’s group utilized a CTF to in situ grow molybdenum sulfide, which served as a HER electrocatalyst requiring only a 93 mV overpotential to achieve a current density of 10 mA cm^−2^ [81]. In 2021, Wang et al. demonstrated outstanding HER electrocatalytic activity by pyrolyzing nitrogen-rich COFs supported by molybdenum and nickel bimetals, achieving a current density of 10 mA cm^−2^ at an overpotential of 104 mV [82].

Recent studies have shed light on the significant role of copper in electrocatalysis. For instance, Iwase et al. developed a sulfur-containing conjugated organic framework CTF with monovalent copper ions, featuring an asymmetric tri-coordination structure and exhibiting a starting potential of 0.88 V as an ORR catalyst [83]. In 2022, Huang et al. engineered a COF with a Salen-Cu structure, which, after pyrolysis, demonstrated an impressive half-wave potential of up to 0.88 V [84]. Rajaopal et al. reported a triazine-based COF (Cu-TTP) that showed remarkable HER catalytic activity after coordinating copper ions and also effectively converted CO_2_ into propylene carbonate with an overpotential of only 115 mV at a current density of 10 mA cm^−2^ [85].

**Figure 6:**
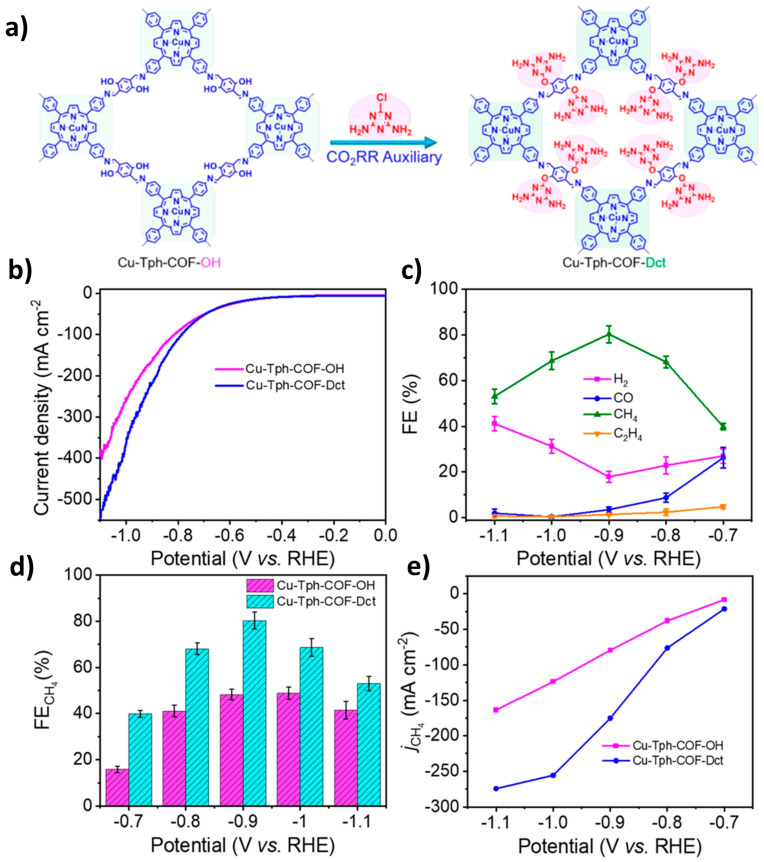
(**a**) The Scheme of the preparation of Cu-Tph-COF-Dct. (**b**) Linear swept voltammetry curves of Cu-Tph-COF-OH and Cu-Tph−COF-Dct. (**c**) FE of Cu-Tph-COF-Dct on CO_2_RR products at different applied potentials. (**d**) FE of CH_4_ at different applied potentials of Cu-Tph-COF-OH and Cu-Tph-COF-Dct. (**e**) Partial current density of CH_4_. Reproduced with permission from ref. [87], Copyright 2021, from Wiley-VCH.

In 2021, Lan’s team designed and synthesized an anthraquinone-rich COF with hollow tubular and fibrous structures, which efficiently reduced carbon dioxide to methane after copper modification, achieving a faradaic efficiency (FE) of 77% for the AAn-COF-Cu catalyst in a three-phase flow cell [86]. In a subsequent study, the same group modified the triazine group in a COF with a copper–porphyrin structure, allowing the COFs to be stripped into nanosheets that achieved a higher FE (about 80%) while reducing CO_2_ to CH_4_, as shown in Figure 6 [87]. Building on this, the same research group synthesized hollow nanospheres based on COF-366 loaded with copper, producing over 84% methane and carbon products [88]. In 2020, Miao et al. designed and synthesized a nitrogen-rich pyridine COF, where its in situ N-heterocyclic carbene could stably load copper nanoparticles and selectively reduce CO_2_ to ethylene, with an FE of 35% [89].

In 2023, our research group developed a three-dimensional metal–organic framework (MCOF) with ctn topology, achieving a total FE of over 50% for various carbon products when utilized as a CO_2_RR electrocatalyst [90]. Wang’s team designed and synthesized COF nanosheets with a sulfonic acid structure, loading them with commercial copper nanoparticles to provide a suitable microenvironment for the directional reduction of CO_2_ to CH_4_, resulting in an FE of over 60% [91]. In 2023, Chen et al. developed a series of hydrophobic COFs with varying alkyl chain lengths for CO_2_RR after copper loading, which improved the FE of C_2_ products to over 80% in a three-phase flow cell [92]. In 2024, Zeng’s group first reported a COF for efficient formic acid synthesis by modulating the electronic state of the copper coordination site. They synthesized a thiophene COF with a copper–porphyrin and electron-rich COF, which could directionally reduce CO_2_ to formic acid with an FE as high as 84% [93].

Iron, as one of the most abundant transition metals, holds great significance in replacing precious metal catalysts [94]. In 2018, Kong et al. designed and synthesized a COF with a bipyridine structure, supported by an Fe-DMSO complex [95]. The core–shell Fe_3_C and the N, S double-doped porous carbon of the FeS composite nanostructure (FeS/Fe_3_C@N-S-C) produced after pyrolysis were utilized as ORR electrocatalysts, exhibiting a half-wave potential of 0.87 V under alkaline conditions. In the same year, Zhu et al. obtained ORR electrocatalysts by pyrolyzing CTFs supported with iron ions [96]. They further investigated how adjusting the ratio of nitrile to benzene during the synthesis of CTFs-based catalysts could yield ORR electrocatalysts with varying catalytic activities. In 2020, Li’s group synthesized a porous carbon nanosphere derived from iron single-atom-supported COFs using an adsorption–pyrolysis strategy, which exhibited a half-wave potential of up to 0.812 V as an ORR electrocatalyst [97]. Similarly, in 2021, Deng’s group synthesized a COFs-derived iron single-atom OER electrocatalyst utilizing a similar method, which demonstrated extraordinary iron–oxygen–nitrogen synergy and required only an overpotential of 290 mV to achieve a current density of 10 mA cm^−2^ [98]. In 2021, Jiang et al. developed an ORR catalyst derived from COFs by pyrolysis, supported by ultra-small iron nanoparticles, exhibiting catalytic activity comparable to that of commercial Pt/C [99]. That same year, Feng’s group developed a series of phthalocyanine-based 2D COFs with different metal coordinations; the phthalocyanine COF with iron coordination displayed an ammonia yield of 33.6 μg h^−1^ mg_cat_^−1^ and an FE of 31.9% as an NRR electrocatalyst [100]. Rojas’ group pyrolyzed pre-loaded iron-rich nitrogen COF in the same year as an ORR electrocatalyst [101]. It was found that the catalysts underwent pickling and secondary heat treatment displayed enhanced ORR activity, indicating that removing large iron aggregates during ORR catalysis improves the performance.

In 2022, Pei et al. developed a COFs-based derivative catalyst with a unique coordination structure of Fe-N_2_-O_2_ [102]. The Fe/Fe_3_C nanoparticles formed after pyrolysis are embedded in a porous carbon backbone and have a half-wave potential of 0.88 V in an alkaline electrolyte when used as ORR electrocatalysts. In the same year, Zhang et al. developed a porous carbon derived from the pyrolysis of iron-salt-loaded CTF for the cathode of microbial fuel cells, exhibiting excellent ORR activity [103]. Liu’s group developed an iron–phthalocyanine-based π-conjugated bibenzyl–azobenzene-linked COF in the same year, showing exceptional ORR catalytic activity with a half-wave potential of an astonishing 0.933 V [104]. Wang et al. prepared a porous Fe-N_x_ nanocluster/carbon ORR electrocatalyst by the pyrolysis of iron-containing CTFs, with a half-wave potential of 0.87 V [105]. Zhu et al. constructed an iron single-atom catalyst derived from iron-doped pyrolysis CTF for bifunctional ORR and OER electrocatalysts [106]. As an ORR electrocatalyst, it demonstrated a half-wave potential of 0.891 V, and as an OER electrocatalyst, it had a potential of 1.543 V at a current density of 10 mA cm^−2^. Zhang’s group designed and constructed a highly conjugated 2D COF based on ferric phthalocyanine in 2023, achieving a half-wave potential of up to 0.92 V as an ORR electrocatalyst [107]. In 2024, Zeng et al. synthesized a 1D COF with an iron-coordinated bipyridine structure, which can reach a current density of 10 mA cm^−2^ at an overpotential of only 225 mV when used as an OER electrocatalyst [108]. In 2024, Zhang’s group designed and synthesized two 3D metalloporphyrin-based COFs. Among them, Fe@NUST-18 with an Fe-N_4_ catalytic site was used as an NRR catalyst, yielding an ammonia productivity of 94.26 μg h^−1^ mg^−1^ and an FE of 18.37% [109]. However, as early as 2016, Cheng’s group reported the synthesis of a three-dimensional hierarchical porous network FeNC catalyst using a simple carbonization method, showing ORR activity comparable to that of commercial Pt/C catalysts, with a half-wave potential of 0.878 V in an alkaline solution [110].

In addition to single-metal-supported catalysts derived from COFs, bimetal-supported COFs have proven to be an effective strategy for enhancing catalytic activity. For example, Wu et al. designed and synthesized iron–cobalt bimetal-supported COFs with bipyridine coordination in 2018 [111]. As an electrocatalyst for the OER, it exhibited a notably low voltage of 1.59 V at a current density of 10 mA cm^−2^, and as a HER catalyst under acidic conditions, it achieved an impressive overpotential of just 260 mV. In 2020, Luo et al. synthesized a sulfonic acid-based COF and utilized a one-pot method to load Cyclen into its pores, resulting in a highly efficient OER electrocatalyst with an overpotential of 276 mV at a current density of 10 mA cm^−2^ [112]. Moreover, they employed an ion exchange strategy to convert the hydrogen sulfonate ions in the same COFs to nickel and iron ions, developing OER electrocatalysts capable of reaching a current density of 10 mA cm^−2^ at an overpotential of 308 mV [113].

That same year, Chen et al. designed a novel COF composite featuring coordination interactions between bis-pyridine and cobalt and iron ions, which displayed an impressive overpotential of only 331 mV at a current density of 10 mA cm^−2^ as an OER electrocatalyst [114]. The team also constructed a nickel–iron-supported OER electrocatalyst using a sulfonic acid COF through ion exchange, achieving an overpotential of 350 mV at the same current density, thereby outperforming monometallic-supported catalysts [115]. Additionally, they developed a modified bipyridine nickel–iron bimetal-supported COF (COF-Bpy@FeNi) that delivered an OER performance with an overpotential of 399 mV at 10 mA cm^−2^ in an alkaline electrolyte [116].

In 2023, Meng et al. leveraged the abundant coordination nitrogen sites within a 3D-COF to coordinate nickel–iron ions, creating a nitrogen-doped porous carbon catalyst encapsulated with a Ni_3_Fe nanoalloy through simple pyrolysis followed by oxidation. This catalyst achieved an overpotential of 328 mV at 10 mA cm^−2^ as an OER electrocatalyst [117]. Similarly, Chen et al. synthesized a pyridine bitriazine COF and fabricated MOx on carbon nanosheets via ion thermal conversion synthesis, resulting in a bifunctional electrocatalyst for both OER and ORR [118]. As an OER electrocatalyst, this material attained a current density of 10 mA cm^−2^ at voltages as low as 1.67 V, while as an ORR electrocatalyst, it reached a high half-wave potential of 0.86 V.

Nickel has shown remarkable potential in the realm of catalysis [119]. In 2016, Vaidhyanathan’s team successfully incorporated nickel nitrate nanoparticles into pre-synthesized benzimidazole coordination frameworks, resulting in an efficient electrocatalyst for the OER, with an overpotential of just 230 mV at a current density of 10 mA cm^−2^ [120]. Building on this, Cao’s team introduced a novel two-dimensional COF in 2020 that featured nickel phthalocyanine components, achieving over 93% selectivity for CO production from CO_2_ [121]. This research progressed further in 2024, revealing a nickel phthalocyanine COF linked via imidazole bonds, which maintained over 90% CO selectivity as a CO_2_RR electrocatalyst across a wide pH range [122]. Similarly, Zeng’s team utilized building blocks based on nickel phthalocyanine to develop COF materials for CO_2_ reduction reactions, resulting in a nickel–nitrogen site COF catalyst that achieved over 95% Faradaic efficiency for CO generation from CO_2_ [123]. Additionally, Xie et al. synthesized nickel phthalocyanine COFs with various push–pull functional groups in 2024, with the NiPc-NH-TFPN-NH_2_ variant displaying over 99% Faradaic efficiency for CO_2_ reduction to CO [124].

Lan’s team designed and synthesized a nickel porphyrin COF combined with ferrocene, showcasing an impressive OER electrocatalytic performance, reaching a current density of 99.6 mA cm^−2^ at 2 V [125]. Moreover, Punniyamoorthy et al. introduced a COF based on β-enaminone linkages that supported various metals, serving as a dual-function catalyst for both HER and OER. The nickel-centered COF demonstrated optimal catalytic activity, requiring an overpotential of only 208 mV to achieve a current density of 10 mA cm^−2^ in acidic electrolytes and 302 mV in alkaline conditions [126].

Additionally, researchers have explored co-doping nickel with other metals to enhance the catalytic activity. In 2024, Li et al. developed a three-dimensional COF incorporating cobalt and nickel porphyrin units as a CO_2_RR electrocatalyst, achieving approximately 95% Faradaic efficiency for converting CO_2_ to CO [127]. Also in 2024, Cao’s team designed and synthesized a conductive MCOF based on nickel phthalocyanine for CO_2_RR, effectively reducing CO_2_ to CO with a Faradaic efficiency of 95% in acidic electrolytes [128]. Furthermore, Veldhuizen et al. created a COF doped with zinc and nickel porphyrins as an efficient CO_2_RR catalyst, attaining a Faradaic efficiency of 79% for CO_2_RR when the two porphyrins were synthesized in a 50% ratio, yielding 69% CO and 10% formic acid [129]. Finally, Huang’s team reported, in 2024, a novel two-dimensional COF containing nickel phthalocyanine and Salen-Co coordination structures as a CO_2_RR electrocatalyst, which efficiently reduced CO_2_ to CO, achieving over 97% Faradaic efficiency [130].

In the field of electrocatalysis, cobalt is one of the most versatile transition metal materials and has been extensively studied within COFs [131]. In 2020, LAN’s research group developed a COF composed of tetrathiafulvalene and cobalt porphyrin, which was converted into covalent organic nanosheets (CONs) with over 90% selectivity. These CONs efficiently reduced carbon dioxide to carbon monoxide within a wide electrochemical window of −0.6 V to −0.9 V [132]. Gong et al. transformed cobalt porphyrin-containing 2D COFs into CONs, named TPPDA-MPor-COFs. These materials effectively reduced CO_2_ to CO within a broad electrochemical window, showing high Faradaic efficiencies of 87% to 90% [133]. Additionally, Ali et al. developed a nitrogen-rich COF with precisely anchored cobalt, converted into CONs as a CO_2_RR electrocatalyst, achieving the efficient reduction of CO_2_ to formic acid with nearly 100% purity in the liquid-phase product by 2022 [134].

**Figure 7:**
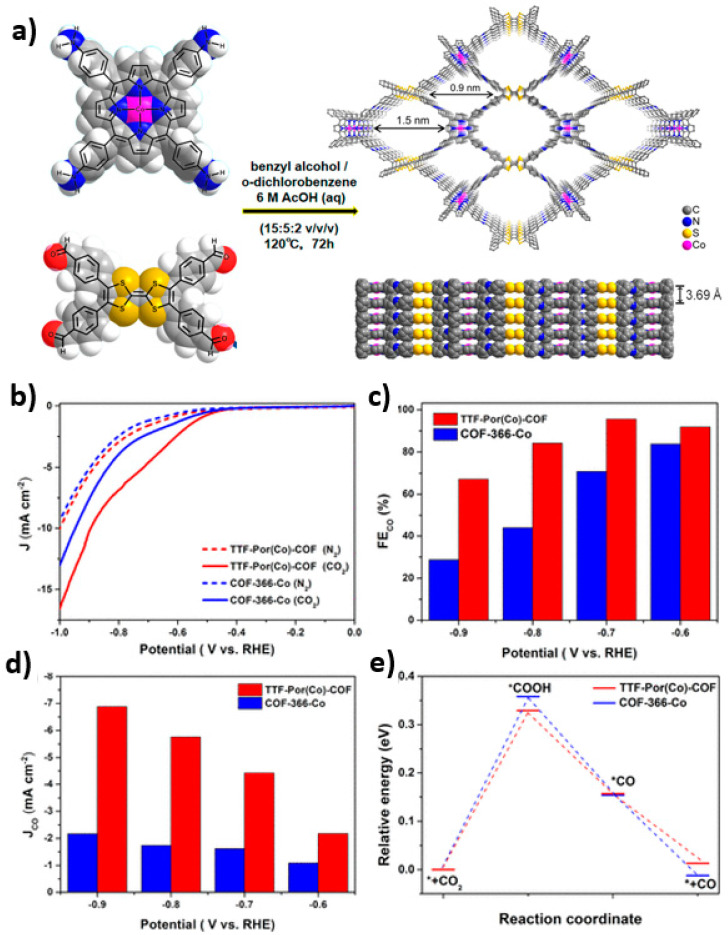
(**a**) Schematic illustration of the synthesis of two-dimensional TTF-Por (Co)-COF. (**b**) LSV curves of 0.5 M KHCO_3_ electrolyte saturated with N_2_ and CO_2_ at a scan rate of 10 mV s^−1^. (**c**) Relationship between FE_CO_ and RHE for TTF-Por (Co)-COF and COF-366-Co in the range of −0.6 to −0.9 V. (**d**) Relationship between the partial current density of CO and RHE for TTF-Por (Co)–COF and COF-366-Co in the range of −0.6 to −0.9 V. (**e**) Relative energy diagram of CO_2_ reduction reaction for TTF-Por (Co)-COF and COF-366-Co at 0 V vs. RHE. Reproduced with permission from ref [136], Copyright 2020, American Chemical Society.

In the same year, Song et al. designed and synthesized cobalt porphyrin-based CONs as an efficient electrocatalyst for the reduction of CO_2_ to CO. In a three-phase flow cell, over 95% FE was reduced to CO, with a stable operating time exceeding 11 h [135]. Continuing in this research direction, Cao’s team designed a 2D COF consisting of tetrathiafulvalene and cobalt porphyrin in 2020, named TTF-Por (Co)-COF, exhibiting an excellent CO_2_RR performance with a reduction rate of carbon monoxide over 95% at a −0.7 V voltage, as shown in Figure 7 [136]. The following year, the same team developed a cobalt porphyrin-based 2D COF with a donor–acceptor (D-A) heterojunction structure [137]. This catalyst efficiently reduced CO_2_ to CO with over 90% FE. In the same study, the research group also reported a 3D cobalt porphyrin COF (3D-Por (Co/H)-COF) that efficiently reduced CO_2_ to CO with over 92% FE [138].

In 2022, Zeng’s research team obtained porous carbon material with a Co-N_5_ coordination pattern by pyrolyzing a cobalt porphyrin COF combined with SP^2^ carbon chains, serving as an efficient CO_2_ reduction electrocatalyst with FE exceeding 90% when converting CO_2_ to CO [139]. Meanwhile, the same team synthesized pyridine-containing COFs in situ on layered double hydroxides (LDH) in a subsequent study. Through a simple pyrolysis method, CoN_2_O_2_ sites were formed, capable of converting carbon dioxide to CO with an FE exceeding 80% [140]. Subsequent research led to a COF (CoPc-EA-COF) with higher electron conductivity and CO_2_ affinity, using tannic acid and phthalocyanine cobalt as COF components. This COF exhibited outstanding CO selectivity as a CO_2_RR electrocatalyst, with an FE exceeding 97% [141].

In a recent study, the team constructed a highly hydrophobic COF containing cobalt phthalocyanine units connected by borate ester linkages. This COF exhibited high CO_2_ affinity and achieved a turnover frequency (TOF) of 1695.3 h^−1^ as a CO_2_RR catalyst [142]. Adding a highlight to advancements in the field, Singh et al. recently introduced hydrophobic alkyl side chains on imine-linked COF and coordinated them with cobalt ions to form a cobalt-based single-atom catalyst (Co-TAPA-OPE). This catalyst demonstrated excellent selectivity in reducing carbon dioxide to ethanol, with an FE of up to 66.8% [143].

**Figure 8:**
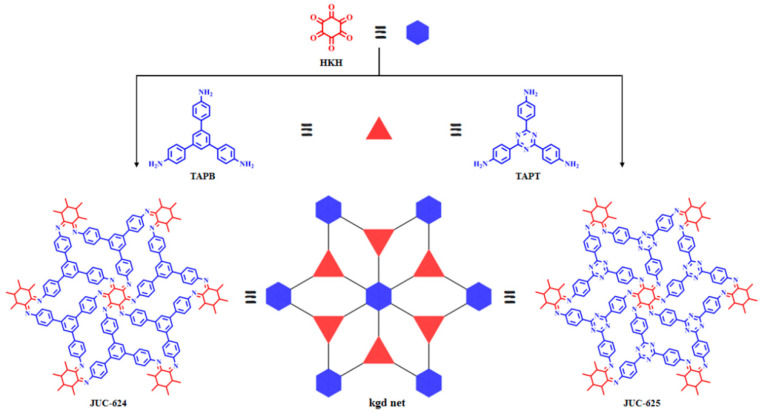
Synthesis and structure of JUC-624 and JUC-625. Reproduced with permission from ref. [129], Copyright 2022, from MDPI.

In 2022, our research team reported the JUC-625, which features triazine structural units and a kgd topology [144]. As shown in Figure 8, the surface of this material is rich in triazine units, providing ample sites for the attachment of cobalt ions. By further reducing cobalt ions to cobalt nanoparticles, we were able to achieve a significant reduction in overpotential for the HER to 146 mV, while achieving a high current density of 10 mA cm^−2^. Also in the same year, Lv et al. reported on a porous carbon material that was loaded with cobalt through the thermal treatment of a triazine-based COF, achieving an impressive current density of 10 mA cm^−2^ with a low overpotential of 303 mV when used as a HER electrocatalyst [145]. Additionally, Zhao et al. developed a three-dimensional COF constructed from spirofluorene and cobalt porphyrin as π-conjugated units [146]. When utilized as a HER electrocatalyst, this COF exhibited a low overpotential of 175 mV, allowing it to achieve a current density of 10 mA cm^−2^.

In 2016, the Banerjee group first designed and synthesized a cobalt-coordinated pyridine-based COF called TpBpy, which demonstrated an overpotential of 400 mV and a current density of 1 mA cm^−2^ as an OER catalyst [147]. Subsequent studies by Zhao et al. involved the synthesis of TpBpy-COF with both micropores and macropores using a polystyrene (PS) template method. When the supported cobalt ions were employed as OER electrocatalysts, the overpotential was reduced to 380 mV at a current density of 10 mA cm^−2^ [148]. In 2021, Ye et al. developed a two-dimensional COF connected by imidazole linkages, which, after modification with cobalt/cobalt oxide nanoparticles, exhibited an OER overpotential of only 278 mV at a current density of 10 mA cm^−2^ [149]. That same year, the Chen group synthesized a nitrogen-rich pyridine-modified COF via the Povarov reaction for OER electrocatalysis [150]. The resulting metal complex (Co@COF-Pyr) showcased an overpotential of 450 mV at a current density of 10 mA cm^−2^.

In 2021, Zhou et al. developed a triazine-based COF with a cobalt loading exceeding 4 wt.%, which served as a bifunctional catalyst for both the ORR and OER [151]. As an ORR catalyst, it displayed a half-wave potential of 0.830 V in alkaline conditions. The Kim group created imidazole-rich COFs doped with cobalt nanoparticles, achieving a high half-wave potential of 0.83 V when used as ORR electrocatalysts [152]. In 2023, Zhang et al. synthesized a cobalt single-atom electrocatalyst, Co-COF-C_4_N, based on COF through theoretical modeling and experimental synthesis, demonstrating an excellent OER performance with an overpotential of 280 mV at 10 mA cm^−2^ [153]. In the same year, Peng’s group designed a three-dimensional porphyrin-based COF with an stp topology, named ZJUT-1. After post-modification with cobalt ions, it exhibited an OER overpotential of 295 mV at a current density of 10 mA cm^−2^ [154]. Concurrently, Wei et al. synthesized a CTF, which, after pyrolysis transformation into cobalt-supported nanoparticle-based porous carbon, displayed a half-wave potential of 0.84 V as an ORR electrocatalyst [155].

In 2024, Wang et al. developed a two-dimensional COF functionalized with thiol groups on the sides. Upon loading with cobalt ions and subsequent thermal treatment, the COF facilitated the in situ growth of Co_9_S_8_, serving as a bifunctional ORR-OER electrocatalyst with a minimal potential difference between the OER and ORR of 0.76 V [156]. Electrochemical ORRs operate through two mechanisms: the two-electron transfer pathway and the four-electron transfer pathway [157]. The former produces hydrogen peroxide, while the latter yields water. Recently, cobalt-containing COFs have shown promise in efficiently synthesizing hydrogen peroxide through electrochemical oxygen reduction. In 2023, Jiang’s group synthesized a two-dimensional COF featuring a sulfur ether chain and cobalt porphyrin structures. This material exhibited superior selectivity for hydrogen peroxide, surpassing 95%, and was able to stably generate a 0.48 wt.% concentration of hydrogen peroxide in a three-phase flow cell for up to 20 h [158]. Subsequently, the same group reported on two other COFs containing cobalt porphyrin and sulfonic acid functional groups, showcasing high stability and selectivity for H_2_O_2_ after 20 h of operation [159]. Additionally, the Chen group synthesized a novel COF (COF-TZ) rich in azide through a continuous ring addition reaction, demonstrating an impressive hydrogen peroxide yield of over 85% during ORRs [160]. In 2019, Xu et al. loaded cobalt ions into the pores of COF-300 and obtained cobalt single-atom catalysts through further thermal treatment [161].

**Figure 9:**
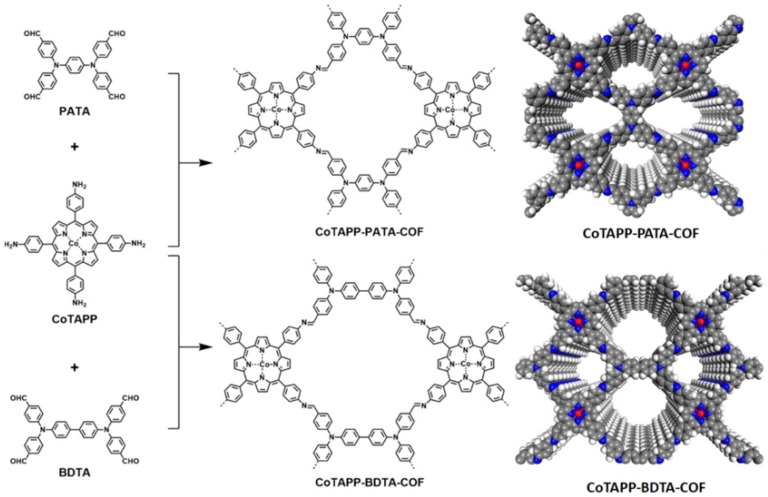
Design and synthesis of catalytic COFs (CoTAPP-PATA-COF and CoTAPP-BDTA-COF) from CoTAPP, PATA, and BDTA, respectively. Reproduced with permission from ref. [162], Copyright 2022, from Wiley-VCH.

Compared with the catalyst without cobalt atoms, the electrocatalytic ORR activity of this catalyst is significantly enhanced, and its half-wave potential and the diffusion limited current density are 0.73 V and 6.1 mA cm^−2^, respectively. In subsequent work, the same group also reported two cobalt-porphyrin-based 2D COFs as OER/ORR bifunctional electrocatalysts as shown in Figure 9 [162].Among them, the half-wave potential of CoTAPP-PATA-COF reaches 0.80 V in ORR and 420 mV at a current density of 10 mV cm^−2^ in OER, as shown in Figure 9. The team also reported in a recent study a DPPS-COF with large layer spacing with a cobalt porphyrin structure [163]. As an advanced ORR electrocatalyst, DPPS-COF has a half-wave potential of 0.85 V, which is superior to commercial Pt/C catalysts. In 2020, Chen et al. in situ grew COF with a bipyridine structure on the surface of silica, and furthermore, the silica template was removed by coordination cobalt post-calcination as an ORR electrocatalyst with initial and half-wave potentials of 0.91 V and 0.83 V, respectively [164]. Zhang’s group reported in 2021 a line-based polyaryl ether-linked COF with unmatched super-stability, acting directly as an ORR electrocatalyst without pyrolysis, and exhibiting a half-wave potential of about 0.73 V [165]. Kim’s group reported at about the same time that a triazine-based COF was prepared by pyrolysis after the coordination of cobalt ions, a porous carbon ORR/OER bifunctional catalyst with cobalt nanoparticle loading [166]. As an ORR electrocatalyst, it has a half-wave potential of 0.85 V, and as an OER electrocatalyst, it has an overpotential of 450 mV at a current density of 10 mA cm^−2^. In 2022, Ren et al. designed and synthesized a 2D COF with a cobalt porphyrin structure with a laterally modified quaternary ammonium salt, which was used as an ORR electrocatalyst with initial and half-wave potentials of 0.88 V and 0.78 V, respectively [167]. In 2022, Song’s group used paleo-coordinated braided COF (COF-112) as an ORR electrocatalyst, with a half-wave potential and diffusion-limited current density of 0.76 V and 5.78 mA cm^−2^, respectively [168]. Liang et al. investigated the electrocatalytic ORR activity of porphyrin-based COFs with different metal coordination [169]. Under a series of different metal coordination conditions, cobalt-coordinated porphyritic COFs have electrocatalytic ORR activity comparable to that of commercial Pt/C catalysts, with a half-wave potential and diffusion-limited current density of 0.66 V and 4.82 mA cm^−2^, respectively. In 2023, Chen’s group designed and synthesized a post-modified method to coordinate different metals in a pre-synthesized 2D COF as a high-efficiency ORR electrocatalyst [170]. Compared with other metals, the COF of cobalt ions has a higher initial potential, half-wave potential, and electron transfer number, which indicates that cobalt is higher than other metals as the active site of ORRs. In 2024, Chen et al. developed a COF-derived cobalt single-atom catalyst with a half-wave potential and diffusion-limited current density of 0.825 V and 5.32 mA cm^−2^ when used as an ORR electrocatalyst [171]. Rani’s group developed a porphyrin and polyoxometallic oxide clusters (POMs)-based COF as a HER electrocatalyst that requires only an overpotential of 75 mV to drive a current density of 10 mA cm^−2^ under alkaline conditions [172]. Compared to cobalt-free catalysts, this catalyst demonstrates significantly enhanced electrocatalytic activity for the ORR, exhibiting a half-wave potential of 0.73 V and a diffusion-limited current density of 6.1 mA cm^−2^.

Transition metal-modified COF-based electrocatalysts offer significant advantages, including enhanced catalytic activity due to the unique electronic structures of transition metals, which provide more active sites for reactions such as ORR, OER, and HER. The electronic structure of the catalyst can be finely tuned by adjusting the oxidation state or coordination environment of the metal, optimizing the catalytic performance and lowering reaction energy barriers. The synergy between the transition metal and the COF framework improves both the stability and activity, with COF’s ordered structure aiding in reactant transport. These catalysts are also more affordable and environmentally friendly, as transition metals like Co, Ni, and Fe are much cheaper than noble metals, and the COF synthesis process is energy-efficient. However, there are challenges, including the potential instability of active sites in the transition metal catalyst, which may lead to site loss or aggregation over time. The synthesis process can be complex, requiring precise control over the metal’s coordination environment, which increases the preparation difficulty and cost. Additionally, transition metal catalysts may show lower selectivity in some reactions, especially in multi-electron transfer processes, leading to side reactions. The limited pore sizes of COFs could also hinder reactant diffusion, affecting the catalytic efficiency. Furthermore, long-term stability under extreme conditions or high current densities may degrade the catalyst’s performance.

### 3.3. Covalent Organic Framework-Based Electrocatalysts for the Recombination of Nano-Carbon Based Materials

In addition to metal materials, nano-carbon–carbon materials, including carbon tubes, can also be used for efficient electrocatalysis by compounding with COFs. On the one hand, nano-carbon materials can improve the intrinsic conductivity of COF materials, and on the other hand, in view of the shortcomings of nano-carbon materials with a single structure and an inability to be designed in a directional manner, COFs can provide efficient active sites for electrocatalysts through its own ingenious design [173]. In this section, we will discuss the research progress of nano-carbon materials and COFs composites as integral electrocatalysts.

#### 3.3.1. COFs-Based Electrocatalysts Based on 1D Carbon Nanomaterials

Carbon nanotubes, as the most common one-dimensional carbon nanomaterial, have shown an outstanding performance in the field of electrocatalysis, mainly due to their high conductivity and tunable surface chemistry [174]. In 2020, Zeng’s research group developed a COF on carbon nanotubes with a structure containing pyridine and coordinated iron and nickel supports [175]. After pyrolysis, this material was used as a bifunctional electrocatalyst for OERs and ORRs. As an ORR electrocatalyst, it achieved a half-wave potential of 0.87 V, while as an OER electrocatalyst, it could achieve a current density of 10 mA cm^−2^ at a voltage of 1.55 V. In this study, the carbon nanotubes provided good mechanical strength and a high specific surface area as the support materials before pyrolysis.

**Figure 10:**
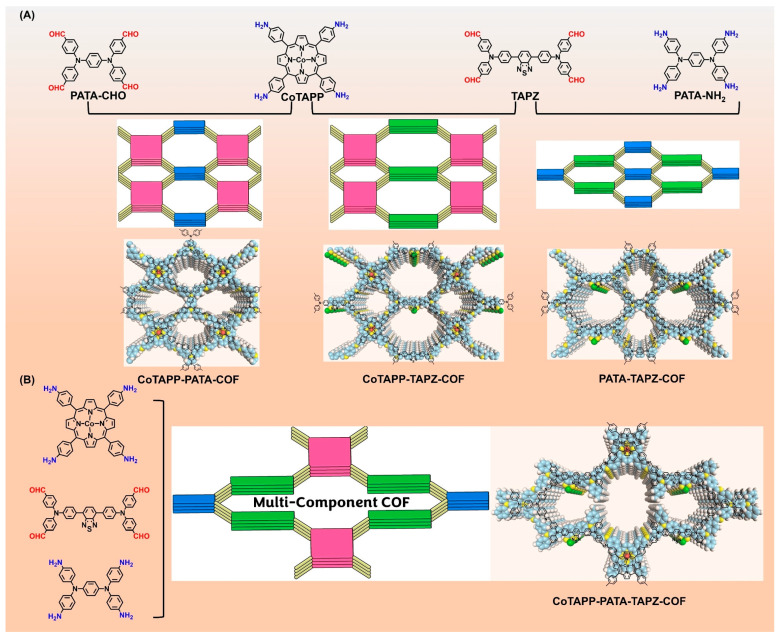
Schematic illustration of chemical structure and synthesis for (**A**) CoTAPP-PATA-COF, CoTAPP-TAPZ-COF, PATA-TAPZ-COF and (**B**) the three-component COF (CoTAPP-PATA-TAPZ-COF) for CO_2_RR. Reproduced with permission from ref. [178], Copyright 2024, from Wiley-VCH.

**Figure 11:**
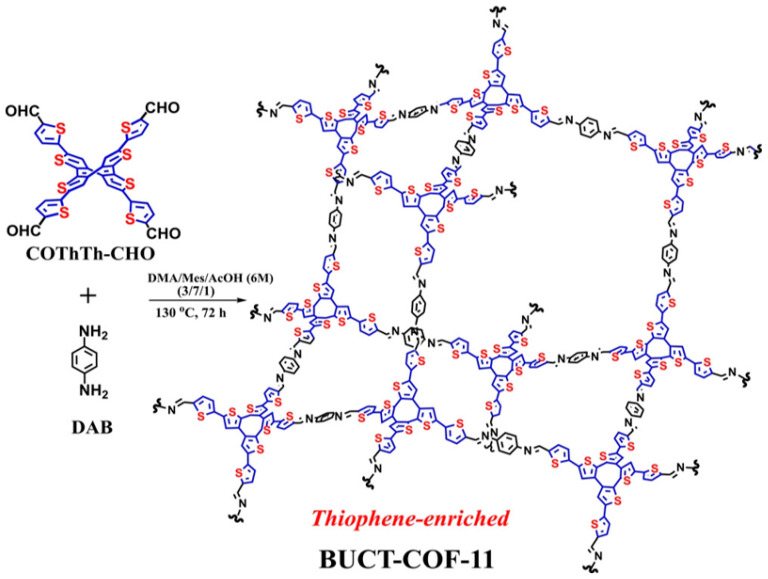
Structure and synthesis of BUCT-COF-11COF. Reproduced with permission from ref. [178], Copyright 2022, from Wiley-VCH.

The same research group subsequently reported that they obtained three-dimensional COF-derived carbons through different templates and immobilized cobalt porphyrins on these carbon materials as electrocatalysts for CO_2_RR [176]. Among them, the COF-derived carbon obtained on the carbon nanotube exhibited the highest CO_2_RR catalytic activity, efficiently reducing carbon dioxide to CO with a current efficiency of 94.5%. Based on this, the same group designed and synthesized a three-component COF with a donor–acceptor–acceptor (D-A-A) structure containing a cobalt porphyrin structure in 2024 [177]. When carbon nanotubes were added as conductive agents, the efficiency of reducing CO_2_ to CO exceeded 92%, as shown in Figure 10. In 2023, Wang et al. designed and synthesized a fully conjugated 3D COF with a saddle-shaped thiophene-rich structure, which was recombined with carbon nanotubes as a high-efficiency ORR electrocatalyst with a half-wave potential of up to 0.72 V, as shown in Figure 11 [178]. In a subsequent study, the same group synthesized a new fully conjugated 3D COF using monomers with the same saddle-shaped thiophene-rich structure [179]. After simple recombination with carbon nanotubes, it could be used as a high-efficiency oxygen reduction electrocatalyst with a selectivity of more than 83% for hydrogen peroxide. Lu et al. achieved the in situ growth of COFs containing cobalt porphyrin functional groups on carbon nanotubes in 2020 [180]. Compared with the simple blending of the two, the carbon monoxide selectivity and FE of this in situ grown COF CO_2_RR electrocatalyst are greatly improved. In 2022, Mo et al. designed and synthesized a COF condensed from cobalt porphyrin and iron-coordinated bipyridine structural units grown in situ on carbon nanotubes [181]. After simple pyrolysis, the ORR catalyst achieved a half-wave potential of 0.862 V as a dual-function catalyst for OERs and ORRs, while the OER electrocatalyst reached a current density of 10 mA cm^−2^ at 1.67 V.

In the same year, Jiang’s group designed and synthesized a heterostructure of nitrogen-rich COF and carbon nanotubes as an ORR electrocatalyst through the department of molecular interface engineering [182]. This work not only changed the path of the ORR from a 2e^−^ process to a 4e-process, but also increased its half-wave potential and diffusion-limiting current density to 0.79 V and 5.5 mA cm^−2^. In the same year, Zhang et al. synthesized a porphyrin-based COF, combining it with four metals (Fe, Co, Ni, and Cu) and achieving in situ growth on carbon nanotubes [183]. Among them, the cobalt coordination exhibited excellent CO_2_RR activity, with a CO reduction efficiency of up to 99.3%. Additionally, Song’s team developed a fully conjugated COF formed by condensing pyridine and cobalt porphyrin on carbon nanotubes [184]. After cobalt ions were coordinated with pyridine, the half-wave potential of this electrocatalyst reached 0.84 V while maintaining an electron transfer number of over 3.8.

Recently, Sun et al. utilized an in situ grown COF with pyridine structures on carbon nanotubes, combined with ruthenium ions, directly serving as an electrocatalyst for HER, achieving an overpotential of 112 mV and a current density of 10 mA cm^−2^ [185]. Li et al. applied a similar method in 2024 to achieve in situ growth of a nickel-supported pyridine COF on carbon nanotubes as a HER electrocatalyst with an overpotential of 160 mV and a current density of 10 mA cm^−2^ [186]. Liu’s team also employed a similar strategy to develop a network of OER electrocatalysts composed of cobalt porphyrin COFs and carbon nanotubes, with an overpotential of 376 mV and a current density of 10 mA cm^−2^ [187]. Meanwhile, Zhang et al. grew a cobalt phthalocyanine COF on carbon nanotubes, which after pyrolysis served as a bifunctional catalyst for ORRs/OERs [188]. As an ORR electrocatalyst, its half-wave potential was 0.83 V, while as an OER electrocatalyst, it could achieve a current density of 10 mA cm^−2^ at 1.70 V.

#### 3.3.2. COFs-Based Electrocatalysts Based on 2D Carbon Nanomaterials

The advancement of graphene-based two-dimensional carbon nanomaterials in electrocatalysis has been ongoing since the introduction of graphene [189]. Like carbon nanotubes, these materials exhibit high conductivity and mechanical strength, with their electrocatalytic potential significantly enhanced when integrated with COFs. In 2017, Jiang’s group made strides by pioneering the in situ growth of conjugated porous framework CTFs on graphene, followed by pyrolysis, resulting in an ORR electrocatalyst with a half-wave potential of 0.83 V and a diffusion-limited current density of 5.64 mA cm^−2^ [190]. Similarly, Zou et al. modified a pyridine side chain on the edge of graphene, linking it to cobalt porphyrin COF through a coordination bond, achieving an ORR electrocatalyst with a half-wave potential of 0.765 V [191]. Roy et al. pyrolyzed a tetrazine-based COF combined with reduced graphene oxide and cobalt salts, producing a cobalt-containing nitrogen-doped porous carbon for bifunctional catalysis in both OERs and ORRs. This catalyst demonstrated a half-wave potential and required a voltage of 1.65 V at a current density of 10 mA cm^−2^ [192].

In 2021, Liu et al. synthesized an ORR electrocatalyst by combining a pre-synthesized iron phthalocyanine COF with reduced graphene oxide. This composite increased the half-wave potential to over 0.8 V compared to the iron phthalocyanine COF alone [193]. That same year, Zhao et al. utilized the confinement effect of a composite formed from triazine COF and graphene to incorporate ruthenium nanoparticles, yielding a HER electrocatalyst with an astonishingly low overpotential of only 42 mV at a current density of 10 mA cm^−2^ [194].

**Figure 12:**
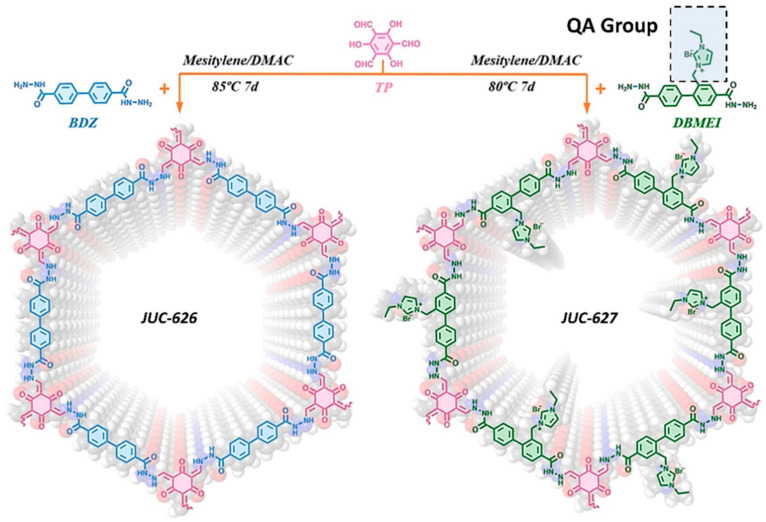
Synthesis and structure of JUC-626 and JUC-627. Reproduced with permission from ref. [197], Copyright 2023, from Elsevier.

In 2022, Zhao’s group developed an aerogel featuring bipyridine functional groups, which was grown in situ on graphene via covalent bonding. After coordinating with cobalt, this material served as an effective catalyst for electrocatalytic water splitting, exhibiting an overpotential of 275 mV as a HER electrocatalyst at a current density of 10 mA cm^−2^ [195]. Additionally, Liu’s group synthesized a nitrogen-rich COF through liquid-phase synthesis and self-assembled it with graphene, utilizing it directly as a HER electrocatalyst without any further treatment. This electrocatalyst presented an extremely low overpotential of just 45 mV at a current density of 10 mA cm^−2^ [196]. The latest composite electrocatalyst involving graphene and COF comprises an OER electrocatalyst formed through the supramolecular self-assembly of graphene and ionic COF nanosheets (JUC-627-NS), as shown in Figure 12, leveraging robust cationic–π interactions. This innovative material can achieve a current density of 10 mA cm^−2^ with an overpotential of only 275 mV [197].

Beyond graphene, C_3_N_4_ represents another two-dimensional nanocarbon material of interest. In 2023, Long’s group anchored iron atoms to a COF containing a triazole building block, followed by calcination after compounding with C_3_N_4_, resulting in a bifunctional electrocatalyst for ORR/OER. This catalyst showcased a half-wave potential of 0.853 V and an overpotential of 346 mV at a current density of 10 mA cm^−2^ [198].

COF-based electrocatalysts modified with graphene and carbon nanotubes offer significant advantages, including enhanced electrical conductivity, a high surface area, and improved structural stability, which boost the catalytic performance in reactions like HER and OER. The combination of graphene and CNTs with COF can increase the number of active sites and promote a better charge transfer, enhancing the overall reaction efficiency. However, challenges exist in their dispersion and integration within the COF matrix as they tend to agglomerate, potentially disrupting the COF’s porous structure and catalytic properties. Additionally, while graphene and CNTs are generally stable, their performance can degrade under extreme conditions, such as high temperatures or strong acidic/basic environments. The functionalization of these materials is also difficult, adding complexity and cost to the catalyst preparation. Despite these drawbacks, graphene and CNTs significantly improve the performance of COF-based electrocatalysts, though scalability and stability under harsh conditions remain areas for further optimization.

### 3.4. Covalent Organic Framework-Based Electrocatalysts for the Recombination of Porous Materials

#### 3.4.1. Covalent Organic Framework-Based Electrocatalysts for the Recombination of MOFs

In the realm of electrocatalysis, MOFs have garnered significant attention due to their high crystallinity, diverse pore structures, and intrinsic metal active sites. MOFs, including ZIFs, possess similar characteristics to COFs, such as designable organic building blocks, high specific surface areas, and well-defined microporous or mesoporous structures. This compatibility allows for the in situ growth of COFs on the surfaces of MOFs through the strategic design of organic building units [199].

In 2017, Zhuang et al. developed a method to achieve the in situ growth of ZIF-67 after modifying COF with benzoic acid [200]. Following pyrolysis, they produced nitrogen-doped porous carbon loaded with cobalt tetroxide nanoparticles, which served as an OER electrocatalyst, exhibiting an overpotential of 330 mV at a current density of 10 mA cm^−2^. Zeng’s group has also focused on developing MOF-COF composite-based electrocatalysts. In 2021, the team grew bipyridyl 2D COFs in situ on the surface of ZIF-8 and subsequently pyrolyzed them in the presence of platinum ions, yielding a nitrogen-doped porous carbon catalyst with platinum–zinc nanoparticles [201]. This catalyst, named Pt–COF@MOF800, demonstrated a half-wave potential of up to 0.85 V, making it a highly efficient catalyst for the ORR. Similarly, in 2022, the same team created porous carbon with CoN_4_O and ZnN_4_ active sites by directly pyrolyzing a non-coordinating metal containing a bipyridine structure grown on ZIF-8 [202]. When employed as a CO_2_RR electrocatalyst, this yielded a faradaic efficiency of 92.6% for the conversion of CO_2_ to CO. That same year, the team re-utilized COF for in situ growth on ZIF-8 and incorporated iron phthalocyanine after carbon removal, achieving an ORR electrocatalyst with a half-wave potential of up to 0.89 V [203]. Likewise, a similar COF was grown on ZIF-67, leading to a core–shell-structured nitrogen-doped porous carbon as a bifunctional ORR/HER electrocatalyst [204]. Under alkaline conditions, this catalyst exhibited an ORR half-wave potential of 0.85 V and a HER overpotential of 160 mV at a current density of 10 mA cm^−2^. The same strategy was employed to grow the same COF on ZIF-67 [205], with a notable difference being the coordination of iridium metal ions onto the bipyridine prior to pyrolysis, forming a core–shell-structured porous carbon catalyst. As an ORR electrocatalyst in an alkaline electrolyte, it displayed a half-wave potential of 0.79 V, while in acidic electrolyte conditions, it had an overpotential of just 48 mV at a current density of 10 mA cm^-^². Additionally, the team recently synthesized a diatomic catalyst based on Fe_2_N_5_P using the same method [206]. This catalyst exhibited an excellent catalytic performance in both acidic and basic ORR processes, with half-wave potentials of 0.75 V and 0.89 V, respectively. In another recent report, the team synthesized a COF in situ on MIL-88A-MOF and coordinated cobalt ions for use as an OER electrocatalyst without undergoing pyrolysis, demonstrating an overpotential of merely 328 mV at a current density of 10 mA cm^−2^ [207].

The same approach was adopted by the same group in another study, where, in 2024, they reported the in situ growth of hetero-organic COFs on the surface of ZIF-8 [208]. They obtained porous carbon with a core–shell structure after coordinating bismuth ions followed by pyrolysis, serving as an ORR electrocatalyst with a half-wave potential of 0.867 V. Zhang et al. initially pyrolyzed ZIF-67 and subsequently grew carbonyl-rich COF in situ on the resulting porous carbon. After secondary pyrolysis, they obtained nitrogen-doped porous carbon as an ORR electrocatalyst, achieving a half-wave potential of 0.841 V [209]. In 2022, Huang’s group developed a COF with thiazole side chains, on which they performed the in situ growth of ZIF-67, resulting in a bifunctional ORR/OER electrocatalyst after pyrolysis, with a potential difference of only 0.75 V [210].

In a similar vein, Zhang et al., in 2022, synthesized MC-X (a nickel- and sulfur-containing MOF) on pre-synthesized COFs, and after pyrolysis, it showed catalytic activities comparable to commercial Pt/C catalysts in alkaline conditions as an ORR electrocatalyst [211]. In the same year, Lan’s team pre-synthesized a copper-coordinated COF (COF-366-OH-Cu) alongside an amino-functionalized MOF, self-assembling the two into a COF@MOF hybrid material [212]. This composite effectively reduced CO_2_ to CH_4_, achieving over 76% efficiency—surpassing that of individually synthesized MOFs and COFs. In 2024, Liu et al. grew a copper–zinc MOF in situ on an electro-spun polyacrylonitrile membrane, then in situ grew a COF-containing bipyridine structure on this base, which, upon pyrolysis, served as a bifunctional ORR/OER catalyst [213]. The catalyst exhibited a half-wave potential of 0.82 V for ORR and an overpotential of 362 mV for OER at a current density of 10 mA cm^−2^.

#### 3.4.2. Covalent Organic Framework-Based Electrocatalysts for the Recombination of MXene

**Figure 13:**
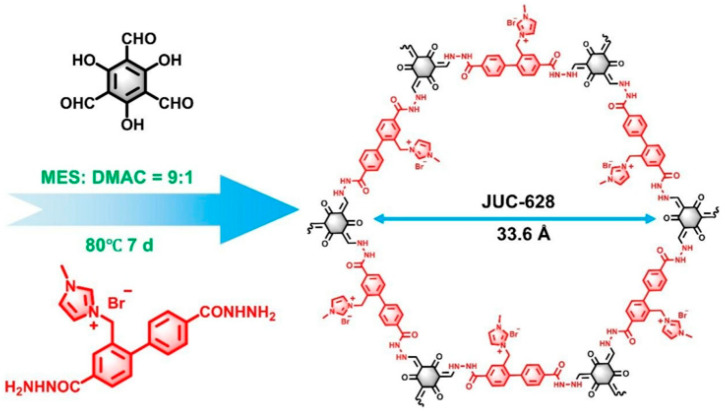
Synthesis and structure of JUC-626 and JUC-627. Reproduced with permission from ref. [220], Copyright 2024, from Elsevier.

As a two-dimensional material with high conductivity and a high specific surface area, MXene has been used in the field of electrocatalysis in recent years [214]. In 2020, Zhu’s group grew COF-LZU-1 in situ as a HER electrocatalyst on pre-synthesized Ni-Ti_3_CNT_x_, which had an overpotential of only 164 mV at a current density of 10 mA cm^−2^ [215]. On this basis, the group also self-assembled COF-LZU-1 on Nb_2_CO_2_ MXene for the first time as an OER, ORR bifunctional electrocatalyst, and the potential difference between the two was only 0.79 V [216]. In 2023, He et al. used the coordination of iron ions on bipyridine COF to modify hydrophobic thiols on the side chain at the same time, and recombined them with MXene as an NRR electrocatalyst [217]. It can efficiently produce NH_3_ at a production rate of 41.8 μg h^−1^ mg_cat_^−1^ and 43.1% FE. Zhou et al. achieved the in situ growth of porphyrin-based COFs on the surface of MXene in 2024, which can reduce CO_2_ to CO at more than 97% FE [218]. Huang’s group also developed an in situ growth of COF-42 on the basis of MXene nanosheets as a HER electrocatalyst [219]. The initial potential of this electrocatalyst is only 19 mV, and the overpotential is only 72 mV at the current density of 10 mA cm^−2^. In a recent study, our group also reported a composite heterostructure of ionic COF nanosheets and MXene assembled by an electrostatic interaction as a HER electrocatalyst (Figure 13), which not only has an overpotential of only 137 mV at a current density of 10 mA cm^−2^, but also realizes the electrolysis of hydrogen production by a COF-based catalyst in seawater for the first time [220].

COF-based electrocatalysts modified with MOF and MXene materials offer significant advantages, including enhanced catalytic activity, improved conductivity, and structural stability. MOFs provide diverse metal centers and organic ligands that optimize the catalytic performance, while MXenes contribute excellent electrical conductivity, lowering the charge transfer resistance. The combination of these materials allows for tunable pore structures and active sites, which can improve the efficiency and selectivity of reactions like ORRs, OERs, and HERs. However, the synthesis of such catalysts is complex, involving precise control over the materials’ structure and compatibility. Stability can be a concern, as MOF metal centers may dissolve under certain conditions and MXenes may degrade in extreme environments. Additionally, the high cost of MOF and MXene synthesis poses a challenge for large-scale production, and potential issues with side reactions or the disruption of COF’s pore structure may the affect performance. Despite these challenges, MOF and MXene-modified COF electrocatalysts hold great promise for improving the performance and versatility of electrocatalysis.

### 3.5. Covalent Organic Framework-Based Electrocatalysts for the Recombination of Organic Molecules and Polymers

Organic molecules, rich in light elements such as boron, nitrogen, phosphorus, and sulfur, can be readily synthesized through the pyrolysis of COF. Additionally, the required doped atoms can be easily incorporated via pyrolysis [221]. Certain organic polymers, like polythiophene and polypyrrole, exhibit high electronic conductivity and can be introduced as conductive agents for COF-based materials. In 2022, Joe’s group pioneered the incorporation of poly(3,4-ethylenedioxythiophene) (PEDOT) into COFs with Salen structures of different metals to form heterostructures [222]. This composite material serves as a HER electrocatalyst, displaying an impressively low overpotential of 150 mV at a current density of 10 mA cm^−2^. In 2024, Yan’s team filled the pores of thiophene-containing COFs with polythiophene to create an ORR electrocatalyst with a half-wave potential of approximately 0.75 V [223]. The introduction of polythiophene further optimizes the electron cloud distribution between COF layers and enhances the electrocatalytic performance of ORR. During the same year, Anwar et al. introduced cellulose nanocrystals as fillers into pre-synthesized polyimide COFs, resulting in HER electrocatalysts with a Tafel slope of about 41 mV dec^−1^ [224]. More recently, Zeng’s group reported a method involving the filling of cobalt porphyrin-based COF pores with phytic acid to serve as a CO_2_RR electrocatalyst [225]. In simulated flue gas conditions, the catalyst demonstrated an ability to reduce CO_2_ to CO with a Faradaic efficiency of over 86%.

Based on the combination of organic small molecules, polymers, and COFs in electrocatalysts, these materials offer notable advantages such as enhanced catalytic activity, stability, and conductivity. The highly ordered pore structure of COFs provides abundant active sites, which, when coupled with the tunable properties of organic small molecules and polymers, improves the charge transfer and reaction efficiency, especially in reactions like ORRs, OERs, and HERs. Moreover, polymers increase the stability of COFs under extreme conditions, and their good conductivity further enhances the overall catalytic performance. However, the synthesis of these composite materials is complex, requiring precise control at multiple stages, which increases the difficulty and cost of production. Additionally, the structural stability of the composites may be compromised under harsh conditions, and the functionalization of organic molecules and polymers remains challenging. Despite these challenges, these COF-based composite electrocatalysts show great potential in energy conversion applications.

## 4. Conclusions and Prospects

Conventional electrocatalysts are typically composed of amorphous porous carbon materials doped with metal-free heteroatoms or metal nanoparticles prepared through pyrolysis. However, the structure of such catalysts is often challenging to define. While the structure and active centers of the catalyst can be finely regulated through complex processes, this often consumes a significant amount of time and resources. In recent years, COF has seen rapid development in the field of electrocatalysis due to its high specific surface area, rich tunable pore structure, and excellent structural stability. Nevertheless, compared to metal materials, COFs exhibit lower electronic conductivity and inferior electrocatalytic activity, making the incorporation of nanomaterials into a COF a key research focus. The combination of nanomaterials with COF-based composite electrocatalysts not only addresses the intrinsic catalytic activity and low electronic conductivity issues of COF catalysts, but also confers the composite catalysts with an outstanding specific surface area and well-defined catalytic active sites, significantly enhancing their application potential as electrocatalysts. This paper summarizes the research progress in almost all COF-based nanocomposite electrocatalysts to date. In over 150 related studies, the structural advantages of various COFs have been emphasized, particularly in the precise anchoring of catalytic active centers and further adjusting the secondary chemical environment through tunable organic building blocks. Furthermore, the multi-level porosity of COF materials, designed with various bonding modes and building blocks, can greatly enhance the mass transfer efficiency of electrocatalysts.

This review specifically discusses COF-based nanocomposite materials as electrocatalysts. Notably, when precious metal nanomaterials (such as platinum, rhodium, ruthenium, iridium, gold, and silver) are combined with COFs, they exhibit remarkable catalytic potential. Meanwhile, non-precious metal nanomaterials (such as cobalt, iron, nickel, copper, and manganese) have gradually become effective substitutes for precious metals and have emerged as the mainstream research direction for advanced nanocomposite electrocatalysts after COF composites. In addition, carbon nanomaterials and conductive polymers are widely used to enhance the electronic conductivity of COF-based electrocatalysts. Despite the promising progress made in the field of electrocatalysis with COF-based nanohybrid and nanocomposite materials, these studies are still in their early stages, facing several key challenges: firstly, how to composite after constructing a clear COF structure, particularly with remaining topological controversies regarding some 3D COFs; secondly, maintaining the COF structure as unchanged when compositing with other nanomaterials is an urgent challenge, especially when some metal-modified COF-based electrocatalysts require coordination under extreme conditions, which often leads to a decrease in COF crystallinity and poses a challenge to COF stability under extreme conditions; lastly, the introduction of nanomaterials not only complicates the in situ analysis of the physical properties and electrochemistry of the catalyst, but also presents a challenge to theoretical simulations, as modeling based solely on COF itself may not accurately reflect the real situation of the catalyst.

Defect engineering, the design of new covalent bonds, and the optimization of interactions between COFs and nanomaterials are key strategies to enhance the catalytic performance. Defect engineering introduces specific defects, such as oxygen or metal vacancies, which create additional active sites and improve electron transfer, enhancing the reaction efficiency. Designing new covalent bonds, such as metal–organic coordination, within the COF framework introduces new catalytic sites, further improving activity by facilitating adsorption and electron transfer. Additionally, optimizing the interactions between COFs and nanomaterials, like metal nanoparticles or graphene, ensures better dispersion and enhances conductivity and stability, contributing to superior catalytic efficiency. These strategies collectively boost COF-based electrocatalysts’ performance by increasing active sites, improving the charge transfer, and optimizing reaction dynamics, making them more effective for energy conversion reactions. In addition, we have compiled the performance of excellent NOF-based electrocatalysts for nanomaterial composites, which are documented in Table A1, Table A2, Table A3, Table A4 and Table A5 of the Appendix A according to their application.

Future research should carefully consider the practical application of COF-based nanomaterial composite electrocatalysts, especially in high current density catalysis, which holds practical significance for industrial production and real-world applications. Studying COF-based nanocomposite electrocatalysts under operational conditions is crucial for understanding the catalytic process and surface–interface reactions and for designing COF-based nanocomposite electrocatalysts with an enhanced catalytic performance. It is hoped that this review will provide valuable insights for the design and development of efficient and highly selective COF-based nanocomposite electrocatalysts.

## Data Availability

Not applicable.

## Appendix A

**Table A1:** HER performance statistics based on metal-free and pyrolysis-free materials.

Compound	Electrolyte	Overpotential at 10 mA cm^−2^ [RHE] (mV)	Tafel Slope (mV dec^−1^)	Doped Nanomaterials	Ref
PtNPs@COF	0.5 M NaHCO_3_	130	-	Pt Nanoparticles	[49]
Pt/COF	1.0 M KOH	23	41	Pt	[50]
NGA-COF@Pt	0.5 M H_2_SO_4_	13	21.88	Pt Single-atom	[51]
Pt@TTB-COF	0.5 M H_2_SO_4_	5	21.6	Pt Single-atom	[52]
NH_2_-COF	0.5 M H_2_SO_4_	19.2	30	Pt/C	[53]
Ru@g-CNx	1.0 M KOH	9	33.2	Ultrafine Ru nanoparticles	[60]
Ru@C_4_N	0.5 M H_2_SO_4_	6	26	Ru	[61]
Rh@CTF-1	0.5 M H_2_SO_4_	57	37	Rh Nanoparticles	[68]
PY-SE-COF-Pd	0.5 M H_2_SO_4_	128	150	Pd^2+^	[69]
CTFs@MoS_2_	0.5 M H_2_SO_4_	93	43	MoS_2_ nanoparticles	[81]
Mo_2_C-MoNi_4_@NC-2	0.5 M H_2_SO_4_	104	75.7	Mo_2_C, MoNi_4_	[82]
BPT-COF-rGO-1	0.5 M H_2_SO_4_	45	53	rGO	[87]
CoNPs@JUC−625	1.0 M KOH	146	186	Co nanoparticles	[144]
TP@VL-COF	0.1 M KOH	75	114	Lindqvist POM, TTris@ZnPor	[172]
c-CNT-0.68@TpBpy-Ru	1.0 M KOH	112	160	Ru^3+^, carbon nanotube	[185]
COF/rGO-Ru	1.0 M KOH	42	46	Ru nanoparticles rGo	[194]
BPT-COF-rGO-3	0.5 M H_2_SO_4_	45	53	rGo	[196]
COF (40%)/Ti_3_C_2_T_x_	0.5 M H_2_SO_4_	72	50	Ti_3_C_2_T_x_	[219]
JUC-628@MXene-66%	1.0 M KOH	137	77.25	Ti_3_C_2_T_x_	[220]

**Table A2:** Part of OER performance statistics based on COFs which doped nanomaterials.

Compound	Electrolyte	Overpotential at 10 mA cm^−2^ [RHE] (mV)	Tafel Slope (mV dec^−1^)	Doped Nanomaterials	Ref
IISERP-COF6_RuO_2_@350	1.0 M KOH	210	67	RuO_2_	[58]
m-CoRu@NC	1.0 M KOH	280	56	CoRu nanoalloy	[59]
Ru@g-CNx	1.0 M KOH	280	49.5	Ru oxide nanoparticles	[60]
Co_0.5_V_0.5_@COF-SO_3_	1.0 M KOH	300	62	Co^2+^, V^3+^	[78]
Fe-SAC@COF	1.0 M KOH	290	40	Iron single-atom	[98]
Fe-TTF-800	0.1 M KOH	315	65	Iron single-atom	[106]
Fe-PTTA-PTDE-COF	1.0 M KOH	225	76	Fe^2+^	[108]
(Cyclen@Ni_0.5_Fe_0.5_)@COF-SO_3_	1.0 M KOH	276	43	Cyclen Ni and Cyclen Fe	[112]
Ni_0.5_Fe_0.5_@COF-SO_3_	1.0 M KOH	308	83	Fe^2+^ Ni^2+^	[113]
O-Ni_3_Fe-N-C	0.1 M KOH	328	52	Ni/Fe bimetal clusters and nanoparticles	[117]
IISERP-COF1_Ni_3_N	1.0 M KOH	230	79	Ni_3_N	[120]
Ni-COF	1.0 M KOH	302	56	Ni^2+^	[126]
Co/CoO@COF	1.0 M KOH	278	80.11	Co/CoO nanoparticles	[149]
Co-COF-C_4_N	0.5 M KOH	280	43	Co^2+^	[153]
ZJUT-1@Co	1.0 M KOH	295	81	Co^2+^	[154]
Ni/Fe-COF@CNT900	0.1 M KOH	320	61	Ni/Fe bimetal clusters and nanoparticles, carbon nanotubes	[175]
JUC-627- NS@G-2	1.0 M KOH	275	137.5	Graphene	[197]

**Table A3:** Part of ORR performance statistics based on COFs which doped nanomaterials.

Compound	Electrolyte	Half-Wave Potential (V)	Number of Electron Transfers (n)	Doped Nanomaterials	Ref
Pt@COF	0.1 M HClO_4_	0.89	3.9–4.0	Pt Nanoparticles	[54]
Pt-OF_800_	0.1 M KOH	0.88	3.95–4.00	Pt subnano- and nanoparticles	[56]
Pt NP@BNC	0.1 M HClO_4_	0.93	4.0	Pt Nanoparticles	[57]
Pd@TB-COF	0.1 M KOH	0.906	3.0	Pd Nanoparticles	[60]
Pd@WTA-COF	0.1 M KOH	0.865	3.92	Pd Nanoparticles	[71]
FeSn_2_@FeSnOx@S–N–C-90	0.1 M KOH	0.88	4.0	FeSn_2_, Fe^3+^, Sn (OH)_x_	[79]
Cu6Sn5@S–N–C-900	0.1 M HClO_4_	0.86	3.9–4.0	Cu,Sn	[80]
Cu NPs/N-C-800	0.1 M KOH	0.88	4.0	Cu Nanoparticles	[84]
FeS/Fe_3_C@N-S-C-800	0.1 M KOH	0.87	3.9	FeS@Fe_3_C nanoparticles	[95]
Fe/Fe_3_C@FeN-C-900	0.1 M KOH	0.88	4.0	Fe^3+^	[102]
FePc-BBL COF	0.1 M KOH	0.933	3.9–4.0	Fe^2+^	[104]
Fe_1.2_NC-0.02-800	0.1 M KOH	0.87	4.0	Fe	[105]
Fe-TTF-800	0.1 M KOH	0.891	3.94–3.96	Iron single-atom	[106]
2D FePc-COF	0.1 M KOH	0.92	4.0	Fe^2+^	[107]
FeNC-900	0.1 M KOH	0.878	3.9–4.0	Fe	[110]
FeCo@NC	0.1 M KOH	0.86	3.55	Co,Fe	[118]
Ni/Fe-COF@CNT900	0.1 M KOH	0.87	3.9–4.0	Ni/Fe bimetal clusters and nanoparticles Carbon nanotubes	[175]
CoFe-COF_bpda_/CNT-700	0.1 M KOH	0.862	4.0	Co,Fe	[181]
COF@MOF800	0.1 M KOH	0.89	3.95–3.99	ZIF-8	[203]
Fe_2_@P-HC	0.1 M HClO_4_	0.89	3.97–3.99	ZIF-8, phytic acid, Fe^2+^	[206]
Bi-COF@MOF-Fe950	0.1 M KOH	0.867	3.81–3.99	Fe-ZIF-8, Bi^3+^	[208]

**Table A4:** Part of CO_2_RR performance statistics based on COFs which doped nanomaterials.

Compound	Electrolyte	Main Products	Faraday Efficiency (%)	Doped Nanomaterials	Ref
AAn-COF-Cu	1.0 M KOH	CH_4_	77	Cu^2+^	[86]
Cu−Tph−COF−Dct	1.0 M KOH	CH_4_	80	Cu^2+^	[87]
COF-366-Cu	1.0 M KOH	CH_4_	68.2	Cu^2+^	[88]
Cu/ICTF50	0.1 M KHCO_3_	C_2_H_4_	35	Cu nanoparticles	[89]
Cu-NUS9	0.5 M K_2_SO_4_, pH = 2	CH_4_	66	Cu nanoparticles	[91]
CuPor-DSDH-COF	0.5 M KHCO_3_	HCOOH	84	Cu^2+^	[93]
0.5NiPc-COF	0.5 M KHCO_3_	CO	95	Ni^2+^	[123]
NiPc-NH-TFPN-NH_2_	0.1 M KHCO_3_	CO	99.6	Ni^2+^	[124]
Co/Ni-TPNB-COF	0.1 M KHCO_3_	CO	95	Co^2+^ Ni^2+^	[127]
NiPc–NiTAA	0.5 M K_2_SO_4_, pH = 2	CO	95	Ni^2+^	[128]
Co−N_2_O_2_NiPc-Salen (Co)	0.5 M KHCO_3_	CO	97	Co^2^, Ni^2+^	[130]
Co-N-COF	1.0 M KHCO_3_	HCOOH	97.4	Co^2+^	[134]
CoTAP-iCON	0.5 M KHCO_3_	CO	95	Co^2+^	[135]
TTF-Por (Co)-COF	0.5 M KHCO_3_	CO	95	Co^2+^	[136]
CoPc-EA-COF	0.5 M KHCO_3_	CO	98.2	Co^2+^	[141]
Co-TAPA-OPE	0.5 M KHCO_3_	C_2_H_5_OH	66.8	Co^2+^	[143]
MWCNT-Por-COF-Cu	0.5 M KHCO_3_	CO	99.3	Cu^2+^, carbon nanotubes	[183]
MXene-Por-COF-Co-7	0.5 M KHCO_3_	CO	97.28	Ti_3_C_2_T_x_, Co^2+^	[218]

**Table A5:** Part of NRR performance statistics based on COFs which doped nanomaterials.

Compound	Electrolyte	NH_3_ Yield Rate	Faraday Efficiency (%)	Doped Nanomaterials	Ref
Ru-TTA-DFP	0.1 M H_2_SO_4_	2 mg h^−1^ mg_cat_^−1^	50	Ru^3+^	[65]
M−HCO−CTF@AuNPs	0.1 M HCl	66.3 μg h^−1^ mg^−1^_cat_	31.4	Au nanoparticles	[67]
Ti-COF	0.05 M HCl	25 μg h^−1^ mg^−1^_cat_	34	Ti^4+^	[74]
TiO_2−x_@COF-Aq	0.1 M HCl	30 μg mg^−1^ h^−1^	16	TiO_2_	[75]
FePc-pz	0.1 m H_2_SO_4_	33.6 μg h^−1^ mg_cat_^−1^	31.9	Fe^2+^	[100]
Fe@NUST-18	0.1 M Na_2_SO_4_	94.26 μg h^−1^ mg^−1^	18.37	Fe^2+^	[109]
COF-Fe/MXene	0.1 M Na_2_SO_4_	41.8 μg h^−1^ mg_cat_^−1^	43.1	Fe^2+^, Ti_3_C_2_T_x_	[217]
